# The leucine zipper domain of the transcriptional repressor Opi1 underlies a signal transduction mechanism regulating lipid synthesis

**DOI:** 10.1016/j.jbc.2023.105417

**Published:** 2023-10-31

**Authors:** J. Pedro Fernández-Murray, Mahtab Tavasoli, Jason Williams, Christopher R. McMaster

**Affiliations:** Department of Pharmacology, Dalhousie University, Halifax, Nova Scotia, Canada

**Keywords:** *Saccharomyces cerevisiae*, lipid, metabolism, membrane biogenesis, transcription, leucine zipper, signal transduction, phosphatidylcholine, phosphatidic acid, Opi1

## Abstract

In *Saccharomyces cerevisiae*, the transcriptional repressor Opi1 regulates the expression of genes involved in phospholipid synthesis responding to the abundance of the phospholipid precursor phosphatidic acid at the endoplasmic reticulum. We report here the identification of the conserved leucine zipper (LZ) domain of Opi1 as a hot spot for gain of function mutations and the characterization of the strongest variant identified, Opi1^N150D^. LZ modeling posits asparagine 150 embedded on the hydrophobic surface of the zipper and specifying dynamic parallel homodimerization by allowing electrostatic bonding across the hydrophobic dimerization interface. Opi1 variants carrying any of the other three ionic residues at amino acid 150 were also repressing. Genetic analyses showed that Opi1^N150D^ variant is dominant, and its phenotype is attenuated when loss of function mutations identified in the other two conserved domains are present in *cis*. We build on the notion that membrane binding facilitates LZ dimerization to antagonize an intramolecular interaction of the zipper necessary for repression. Dissecting Opi1 protein in three polypeptides containing each conserved region, we performed *in vitro* analyses to explore interdomain interactions. An Opi1^1-190^ probe interacted with Opi1^291-404^, the C terminus that bears the activator interacting domain (AID). LZ or AID loss of function mutations attenuated the interaction of the probes but was unaffected by the N150D mutation. We propose a model for Opi1 signal transduction whereby synergy between membrane-binding events and LZ dimerization antagonizes intramolecular LZ–AID interaction and transcriptional repression.

Opi1 from *Saccharomyces cerevisiae* is the founding member of a transcriptional repressor protein family defined by the array of three domains: the leucine zipper (LZ), the uncharacterized conserved region (UCR), and the activator interacting domain (AID) (Figs. 1*A* and S1). This tripartite array is present at the C terminus of polypeptides of variable length across the Opi1 protein family. A role for Opi1 proteins as transcriptional repressors has been revealed from studies in *S*. *cerevisiae* and other three saccharomycetes: *Candida glabrata, Yarrowia lipolytica*, and *Candida albicans* (1, 2, 3, 4, 5, 6). Most of the knowledge accrued about Opi1 and its functional partners, the transcriptional activators Ino2 and Ino4, comes from studies in *S*. *cerevisiae* unraveling inositol availability, phospholipid (PL) synthesis, and lipid homeostasis (Fig. 1*B*) (1, 2, 6). The so-called Henry regulon is a repressor loop autoregulatory circuit to which the *INO1* gene, encoding the enzyme catalyzing the rate-limiting step of *de novo* inositol synthesis, is highly wired. Depending on the cellular demand for inositol, a broad growth phenotype is associated with the regulation of *INO1* expression: from *ino* phenotype, scored as growth impairment in the absence of exogenous inositol, to *opi* (*o*ver*p*roduction of *i*nositol) phenotype, where inositol biosynthesis exceeds cellular needs. Ino2 and Ino4 belong to the basic helix-loop-helix family of transcriptional activators, and as a heterodimer, they drive transcription upon binding to *cis*-regulatory elements at the promoter of targeted genes. Opi1 is the transducer of the regulon. Opi1 interacts through its AID with Ino2 preventing transcription, with mutants bearing truncations of this domain or point mutations affecting conserved residues defective in Opi1-repressing function (7, 8, 9, 10). Opi1-repressing activity over Ino2/Ino4 targeted genes is antagonized by its association with the endoplasmic reticulum (ER).

**Figure 1:**
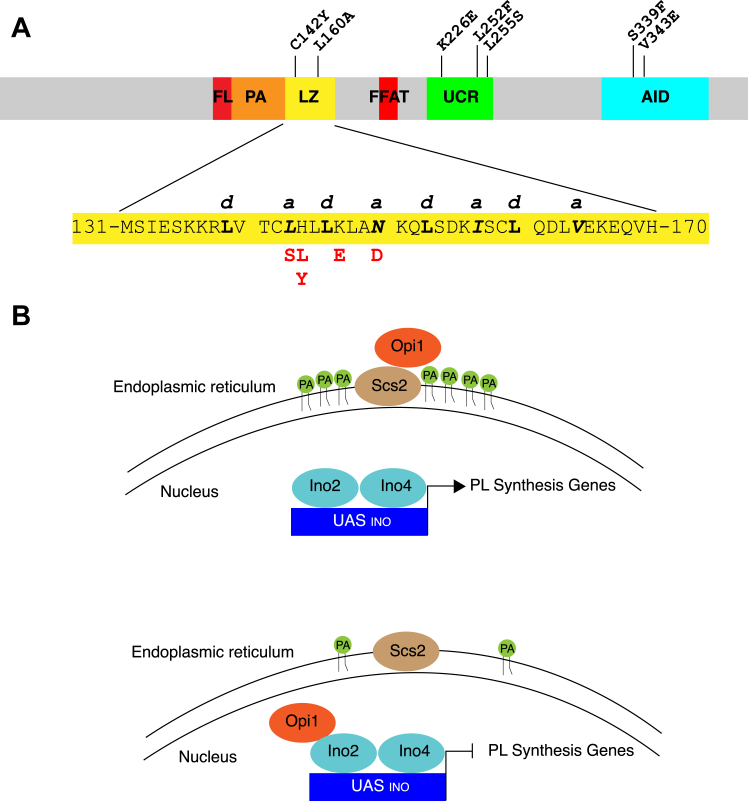
**Domain architecture of Opi1.***A*, the conserved domains leucine zipper (LZ) (aa. 139–166), uncharacterized conserved region (UCR) (aa. 220–256), and activator interacting domain (AID) (aa. 321–379) are indicated in *yellow*, *green*, and *blue*, respectively. The AID of Opi1 is known to physically interact with Ino2 to prevent transcription. The functions of the LZ and UCR domains of Opi1 are not known. The phosphatidic acid (PA)-binding region (aa. 109–138) is indicated in *orange*, and the two phenylalanines in an acidic tract (FFAT) (aa. 193–204) and the FFAT-like (FL) (aa. 98–108) motifs that bind to the ER-resident proteins Scs2 and Scs22 are indicated in *red*. Previously known loss of function (LOF) mutations in Opi1 further analyzed in this study are indicated above the linear representation of Opi1 and are C142Y, L160A, K226E, L252F, L255S, S339F, and V343E. The newly identified gain of function (GOF) mutations in Opi1 reported in this study (L143S, H144L, H144Y, K147E, and N150D) are all in the LZ and are denoted in *red* below the amino acid sequence of the LZ. Heptad repeat residues for LZ modeling are named *a* to *g*. Leucine residues occupying the *d* positions of the heptad repeats of the Opi1 LZ are bolded whereas residues occupying the *a* positions are italicized and bolded, respectively. *B*, current model for Opi1 regulation of the transcription of phospholipid (PL) biosynthetic genes in *Saccharomyces cerevisiae*. Opi1 associates with the integral ER protein Scs2 as well as PA. When PA levels are high (such as when the PL precursor inositol is limiting), Opi1 is retained in the ER allowing Ino2/Ino4 to drive the transcription of PL biosynthetic genes *via* the inositol upstream activation sequence (UAS_INO_) present in their promoters. When PA levels are low (which occurs when inositol is in excess), Opi1 is released from the ER where it subsequently enters the nucleus and binds to Ino2, resulting in the repression of PL gene transcription. ER, endoplasmic reticulum; PL, phospholipid.

Opi1 recruitment to the ER depends on the binding to phosphatidic acid (PA) and to the integral ER protein Scs2 (11, 12, 13, 14, 15). The PA-binding motif features an amphipathic helix with two basic lysine-rich grips that confer specificity towards the anionic head group of PA and a tyrosine residue enabling interfacial hydrophobic interaction (14). Scs2 and its paralog Scs22 are the yeast members of the eukaryotic VAP family (16, 17). VAP proteins are binding partners for soluble proteins involved in lipid homeostasis and organization of membrane contact sites (12, 13, 18, 19, 20). In addition of its well-characterized VAP-binding two phenylalanines in an acidic track (FFAT) motif, Opi1 features a putative second FFAT-like (FL) motif (21) (Figs. 1*A* and S1). Importantly, beyond *INO1* gene regulation, the Henry regulon contributes to direct the lipid flux towards membrane synthesis or storage in response to cellular inputs. The role of the regulon to maintain lipid homeostasis is illustrated by the extreme phenotypes associated with its mutants. Hampering the synthesis of PL by mutating *INO2* or *INO4* leads to supersized cytosolic and nuclear lipid droplets formation (22, 23, 24), whereas preventing Opi1 repression results in ER membrane expansion (25, 26, 27, 28). Most of the signals affecting the output of the regulon impinge on Opi1 function modulating its repressing activity over Ino2/Ino4 targeted genes. The dependance on PA binding and Scs2 interaction for Opi1 membrane recruitment and consequent gene derepression connects the local availability of the precursor PA with the metabolic output of the regulon: membrane synthesis. Until this report, only variants of Opi1 with reduced affinity for PA or for Scs2 were known to repress transcription, eliciting an *ino* phenotype (11, 12, 13, 14, 18, 29).

In this study, we sought to further understand the mechanisms by which Opi1 transduces membrane signals into gene expression regulation. We generated a collection of *OPI1* alleles and screened for those that were resistant to derepressing conditions and identified the LZ as a hot spot for gain of function mutations. The characterization of the variant Opi1^N150D^ reported here, affecting a canonical asparagine residue of the LZ, implies that LZ-mediated homodimerization attenuates Opi1 repression. We propose a model whereby membrane binding through the PA-binding region and FFAT motifs facilitates LZ dimerization, antagonizing the intramolecular interaction of the LZ to the AID, required for Opi1 transcriptional repression of lipid-synthesizing genes.

## Results

### Identification of the LZ as a hotspot for repressing variants of Opi1

In *S. cerevisiae*, phosphatidylcholine (PC) is the end-product for PL synthesis through both CDP-diacylglycerol and CDP-choline branches, working as a sink for phosphatidylserine and phosphatidylethanolamine metabolism (30). The exogenous level of choline coordinates the partitioning of PC synthesis between these two pathways; augmenting the level of exogenous choline increases the contribution of the Kennedy pathway to PC synthesis (31). PC homeostasis impacts on Opi1 regulation. Increased PC synthesis by choline supplementation elicits or exacerbates the *ino* phenotype of many yeast mutants (32), and for the few tested, blocking the synthesis of PC through the CDP-choline pathway prevents the deleterious effect of choline (17, 29, 33). Beyond PC synthesis, PC turnover has also been demonstrated to regulate Opi1 localization and transcriptional repression. Nte1 is a conserved phospholipase B localized at the ER that hydrolyzes PC into glycerophosphocholine (GPC) and two free fatty acids (FFA) (34). In *nte1*Δ cells, Opi1 preferentially localizes to nucleoplasm and elicits a choline-dependent *ino* phenotype (33, 35), while increasing *NTE1* gene dosage alleviated the *ino* phenotype of mutants defective in diverse cellular function including *scs2*Δ, *scs3*Δ, *ire1*Δ*, snf1*Δ, *snf2*Δ, *ino80*Δ, and *mpk1*Δ cells (33, 35). These genes, or genes affecting the same signaling pathways or macromolecular complexes, are hypostatic to *OPI1* regarding their *ino* phenotype (33, 35, 36, 37, 38), suggesting that the membrane changes introduced by Nte1 alleviate Opi1 repression. Indeed, increasing *NTE1* gene dosage leads to the derepression of well-known gene reporters of the Henry regulon for inositol grown cells (33).

**Figure 2:**
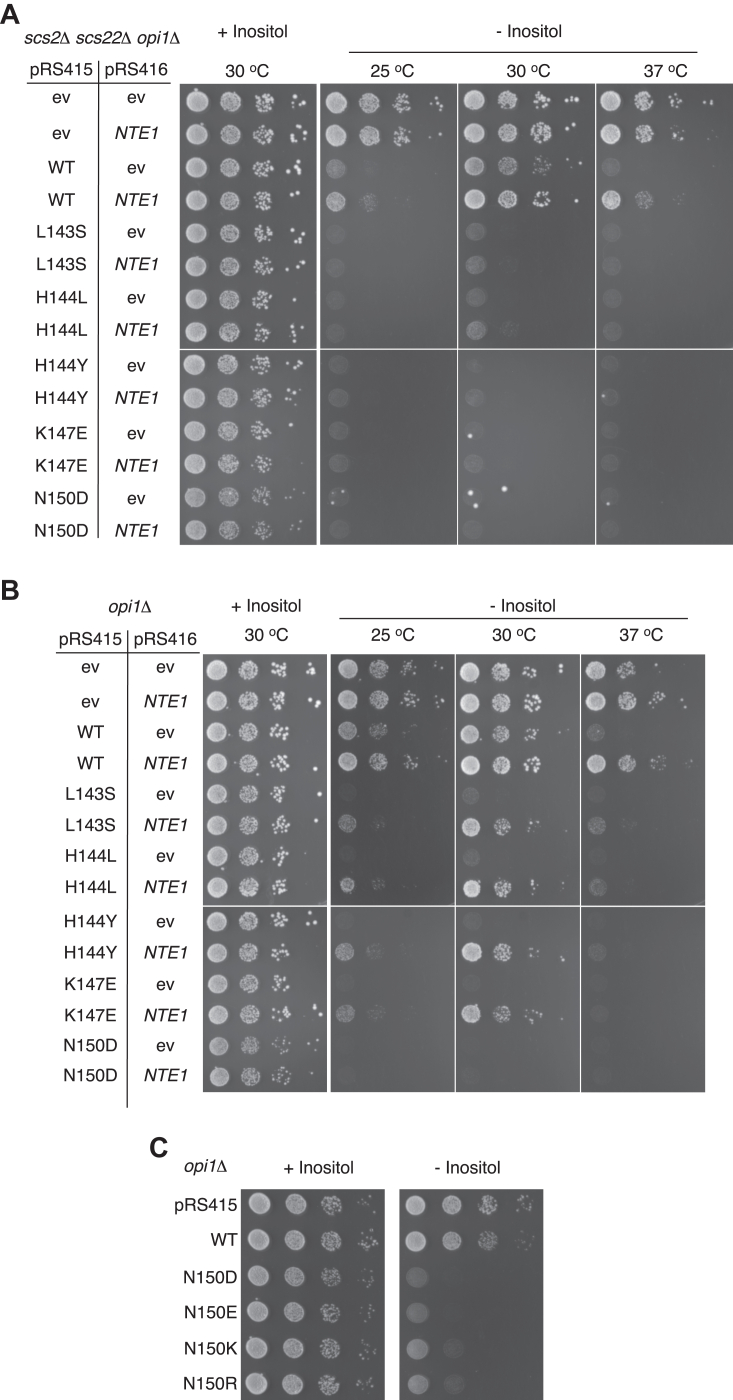
**Phenotypes of the newly identified Opi1 gain of function mutants.***A* and *B*, GOF Opi1 ^L143S^, Opi1^H144L^, Opi1^H144Y^, Opi1^K147E^, and Opi1^N150D^ variants were expressed from a pRS415 vector in *scs2*Δ *scs22*Δ *opi1*Δ (*A*) and *opi1*Δ (*B*) cells transformed with a pRS416 vector carrying or not the *NTE1* gene. *OPI1* and *NTE1* genes are under control of their own promoters; the pRS series of vectors are present in yeast at ∼2–3 copies per cell. Transformed cells were grown in synthetic selective media containing 0.2 mM inositol for 16 h at 30 °C. Cells were thoroughly washed and spotted as a 10-fold serial dilution at an initial cell density of A_660 nm_ of 0.4 onto synthetic selective solid media containing or not 0.2 mM inositol. Plates were incubated at the indicated temperatures for 3 days. *C*, ionic residues at the position *a* 150 of Opi1 LZ elicit *ino* phenotype. *opi1*Δ cells transformed with a pRS415 vector encoding or not the indicated *OPI1* allelic variants were grown in synthetic selective media containing 0.2 mM inositol for 16 h at 30 °C. Cells were thoroughly washed and spotted as a 10-fold serial dilution at an initial cell density of A_660 nm_ of 0.4 onto synthetic selective solid media containing or not 0.2 mM inositol. Plates were incubated at 30 °C for 3 days. GOF, gain of function; LZ, leucine zipper.

Aiming to identify structural elements of Opi1 responding to Nte1-mediated lipid metabolic changes, we performed a genetic screen for Opi1 allelic variants that were refractory to Nte1-increased expression. Considering the direct role of Scs2 and its paralog Scs22 towards Opi1 membrane association, we selected a *scs2*Δ *scs22*Δ background to perform the selection for Opi1 variants overcoming the Nte1 beneficial effect and reimposing the *ino* phenotype characteristic of the *scs2*Δ *scs22*Δ strain. To do so, we amplified the *OPI1* ORF under error-prone conditions and transformed the resulting amplicons, along with a gapped plasmid bearing the promoter and terminator regions of the *OPI1* gene at its free ends, into *opi1*Δ *scs2*Δ *scs22*Δ cells carrying an extra copy of *NTE1* on a plasmid. Yeast cells, through endogenous homologous recombination of these two linear DNA fragments, enabled repair of replicating and selectable plasmids. Transformed cells were plated onto selective inositol-rich media and cells containing repaired plasmids were grown into colonies at 30 °C. Colonies were replica plated onto selective inositol-free media and cultured at 37 °C, a temperature known to exacerbate the *ino* phenotype of *scs2*Δ *scs22*Δ cells and its alleviation mediated by Nte1. As it is shown in Figure 2*A*, increased *NTE1* gene dosage improved the performance of *scs2*Δ *scs22*Δ cells carrying the WT allele of *OPI1* when cells were challenged at 37 °C in the absence of inositol. Colonies that performed poorly were selected and their phenotype reassessed/confirmed by serial dilution plate growth assays. Plasmids recovered from the selected clones were transformed back into *opi1*Δ *scs2*Δ *scs22*Δ pRS416-*NTE1* strain and rescreened to ensure that the *ino* phenotype was imparted by a plasmid borne character. From ∼50,000 colonies screened, seven clones were selected for their ability to reverse the inositol proficiency of a *scs2*Δ *scs22*Δ strain expressing plasmid born *NTE1*. Sequencing of the *OPI1* ORF from the isolated plasmids, followed by restriction fragment swapping, and finally introduction of single amino acid changes by site-directed mutagenesis revealed that L143S, H144L, H144Y, K147E, and N150D were necessary and sufficient to overcome Nte1 beneficial effect and reimpose the *ino* phenotype (Figs. 1*A* and 2*A*). Interestingly, all the Opi1 mutants derived from this screen were in the LZ domain.

Further characterization of each of the Opi1 LZ variants isolated was undertaken. Whereas *scs2*Δ *scs22*Δ cells expressing Opi1^WT^ grew at 37 °C when carrying the pRS416-*NTE1* plasmid, cells expressing any of the mutated variants performed poorly under the same condition. However, these variants imposed a growth limitation for *scs2*Δ *scs22*Δ cells independently of *NTE1* dose when growth was assessed at 30 °C (Fig. 2*A*). We re-assessed the susceptibility of these variants to increased Nte1 expression in the presence of Scs2/Scs22. The *OPI1* alleles were expressed in an *opi1*Δ strain carrying or not *NTE1* gene on a plasmid and their growth phenotype examined (Fig. 2*B*). Whereas the increased expression of Nte1 alleviated the *ino* phenotype elicited by Opi1 variants carrying the mutations L143S, H144L, H144Y, or K147E, it was unable to reverse the growth impairment upon the expression of Opi1^N150D^.

Consistent with its conservation across the Opi1 protein family, the LZ of Opi1 is known to be a spot for loss of function (LOF) mutations. Opi1^C142Y^ was isolated through a genetic screen (10) and Opi1^L160A^ was generated by design replacing a leucine residue of the zipper (39) (Figs. 1*A* and S1). The present screen reveals the LZ as a hot spot for repressing variants. The differential sensitivity of these new alleles to Nte1-mediated membrane changes depending on the presence of Scs2/Scs22 and the broad phenotypic spectrum of the LZ variants suggest that the LZ plays a dynamic role supporting Opi1 function.

### Characterization of the Opi1^N150D^ variant

The LZ of Opi1 exhibits characteristic elements of zippers mediating dynamic parallel homodimerization. It is composed by three heptad repeats with the uniform presence of leucine residues at the *d* positions and branched aliphatic residues like isoleucine or valine at the *a* positions of the heptads, a signature for parallel dimerization (Fig. 1*A*) (40, 41, 42, 43, 44). Recently, it was determined that the N terminus of Opi1, which contains the LZ motif, underwent dimer-monomer transition in a concentration-dependent manner (14). Importantly, the asparagine residue 150 is located at the *a* position of the central heptad which is thought to be a critical determinant for dynamic parallel homodimerization, contributing both to the specificity and affinity of the dimer (45, 46, 47). The mutation N150D, adding a negative charge to this position, would be predicted to reduce the stability of an Opi1 dimer (45, 46, 47). Consistent with the notion that N150D imposes charge repulsion reducing LZ dimerization of Opi1, the expression of negatively charged variant Opi1^N150E^ or the positively charged variants Opi1^N150K^ and Opi1^N150R^ in an *opi1*Δ strain all impaired growth under inositol limitation (Fig. 2*C*). We assessed the phenotype arising from the N150I mutation. Isoleucine is the most frequent residue found at *a* positions of canonical zippers. Contrarily to the charged variants, Opi1^N150I^ conferred a growth phenotype very similar to the WT allele across conditions of increasing inositol demand (Fig. S2), confirming that the ionic character of the residue 150 increases Opi1 repression. We also explored the notion that charge cancellation could allow dimer formation and consequent derepression. The co-expression of Opi1^N150K^ or Opi1^N150R^ together with Opi1^N150D^ in an *opi1*Δ strain did not attenuate the growth restriction under inositol limitation (Fig. S3), suggesting that salt bridge formation between opposite charged residues at the *a* 150 position would not support Opi1 parallel LZ dimerization.

**Figure 3:**
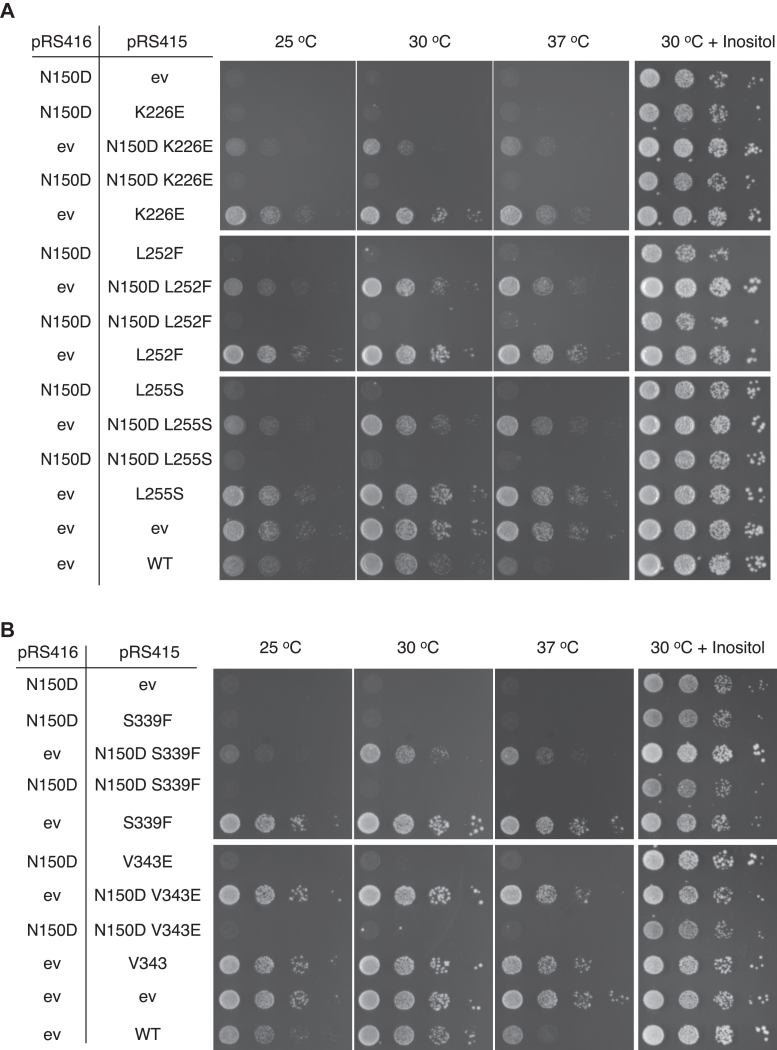
**The Opi1**^**N150D**^**gain of function variant is dominant only in *trans*.***A* and *B*, LOF mutations K226E, L252F, and L255S associated with the UCR (*A*) and S339F and V343E associated with the AID (*B*) were assessed in *trans* and *cis* configurations with the mutation N150D. LOF variants carrying or not the N150D mutation in the same polypeptide were expressed from a pRS415 vector, whereas Opi1^N150D^ was simultaneously expressed or not from a pRS416 vector into *opi1*Δ cells. Transformed cells were grown in synthetic selective media containing 0.2 mM inositol for 16 h at 30 °C. Cells were thoroughly washed and spotted as a 10-fold serial dilution at an initial cell density of A_660 nm_ of 0.4 onto synthetic selective solid media containing or not 0.2 mM inositol. Plates were incubated at 25 °C, 30 °C, and 37 °C for 2 days. AID, activator interacting domain; ev, empty vector; LOF, loss of function; UCR, uncharacterized conserved region.

If LZ dimerization antagonizes monomerization and repression, an Opi1 variant unable to dimerize would be dominant. We explored this notion by co-expressing different alleles of *OPI1* from two plasmids in an *opi1*Δ strain and assaying growth under inositol limitation. Three previously known LOF mutations identified in the UCR: K226E, L252F, and L255S and two identified in the AID: S339F and V343E (9, 10) (Figs. 1*A* and S1) were analyzed in *trans* and *cis* configurations to the N150D mutation identified here. These LOF variants performed like *opi1*Δ and better than WT when inositol-deprived cells were exposed to suboptimal temperatures (Fig. 3, *A* and *B*). The phenotype imparted by Opi1^N150D^ was not prevented by the co-expression in *trans* of any of these variants. However, it was abrogated or attenuated when these mutations were present in *cis* of the N150D mutation, supporting the notion that a variant unable to dimerize is dominant but implying that mere monomerization does not confer repression. The functional integrity of either the UCR or AID-conserved domains is necessary for Opi1 to acquire repression.

**Figure 4:**
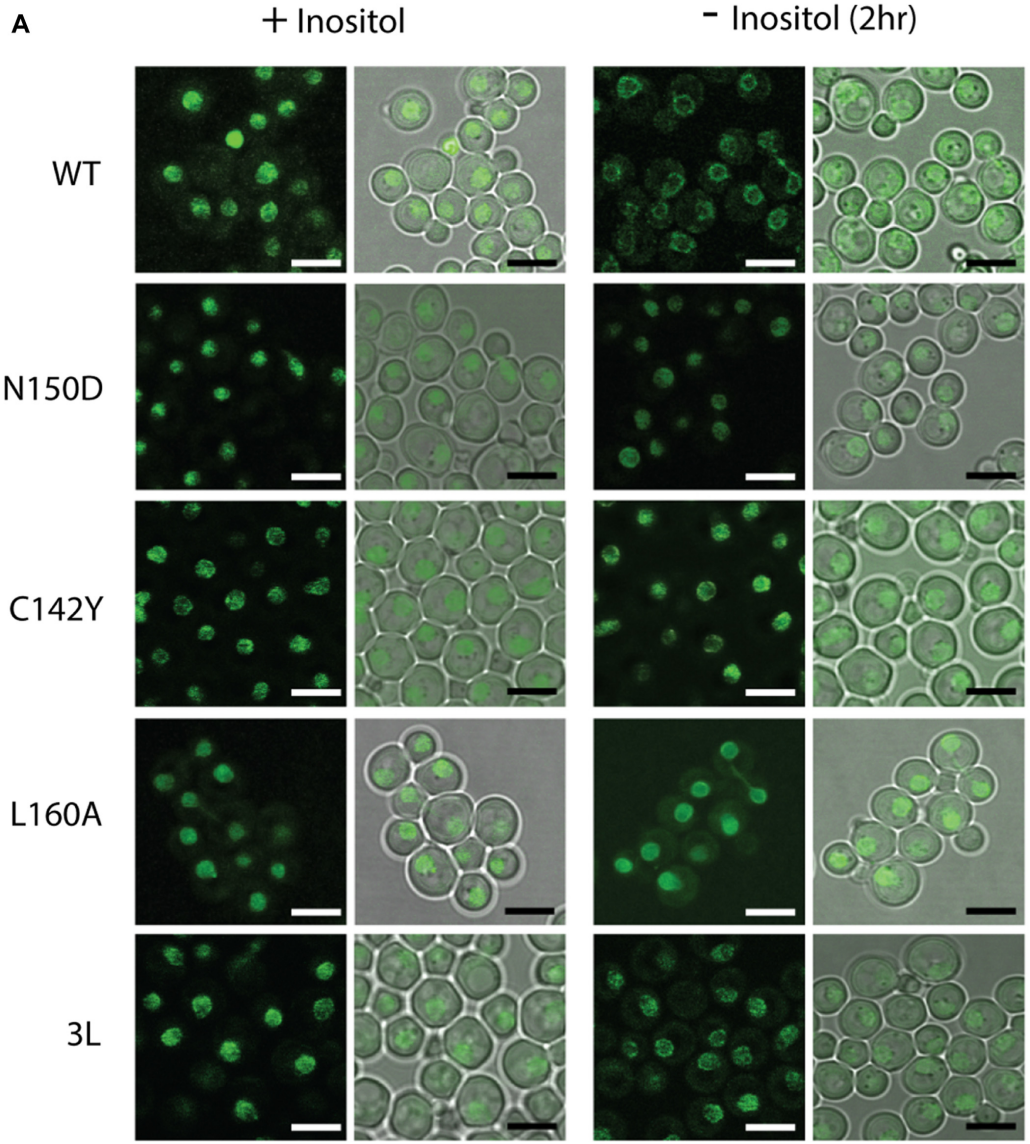

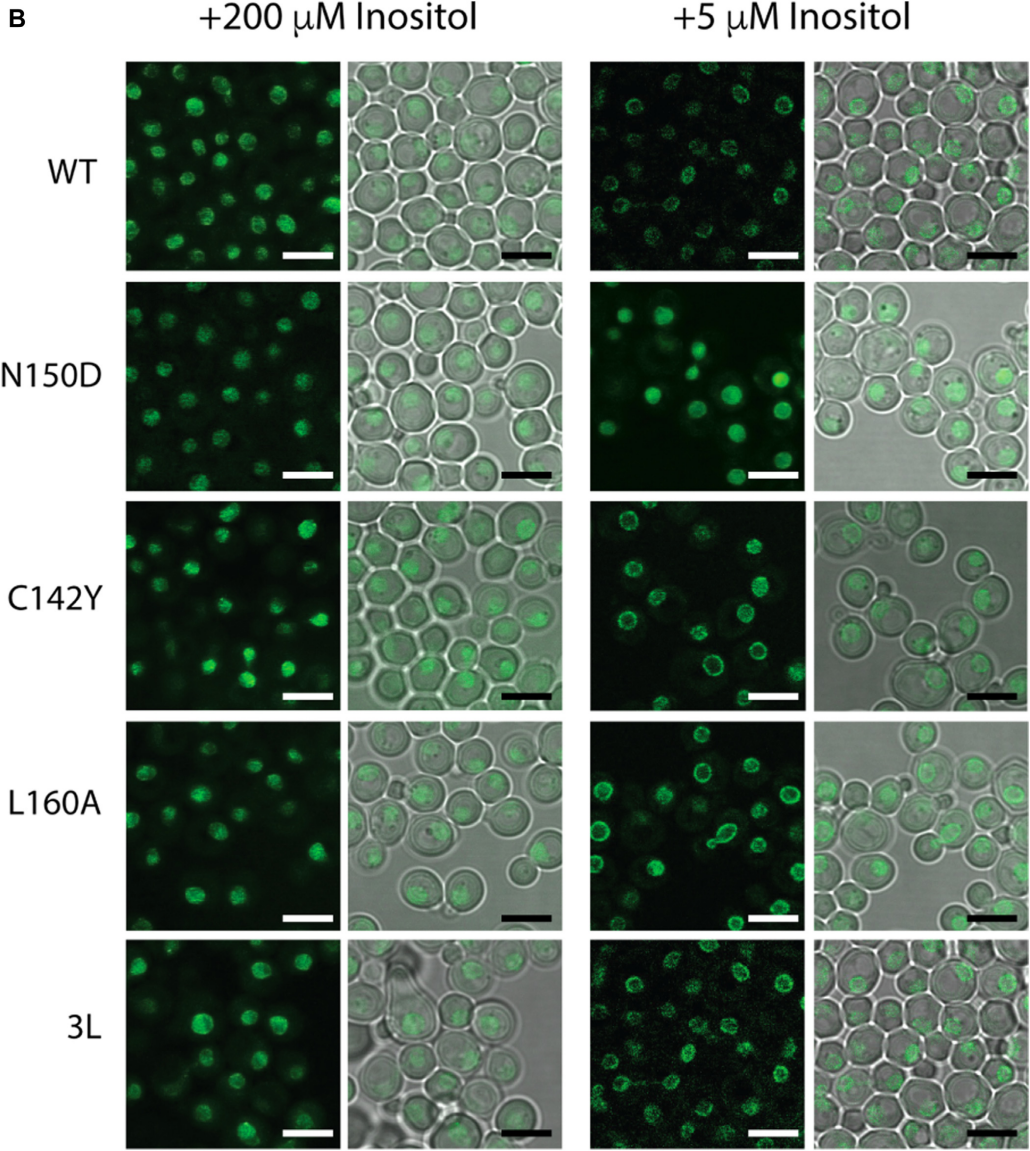
**Persistent nucleoplasmic localization of the Opi1**^**N150D**^**variant.***A*, cells expressing Opi1^WT^, Opi1^N150D^ and LOF Opi1^C142Y^, Opi1^L160A^ and Opi1^L146A, L153S, L160A (or 3L)^ as C-terminal m-GFP fusion from *OPI1* chromosomal locus were grown into log phase in defined media containing 0.2 mM inositol. Cells were thoroughly washed and cultivated for 2 h in define media containing or not 0.2 mM inositol. Live cell images were acquired by confocal microscopy. *B*, yeast *ino1*Δ cells expressing the indicated LZ variants as C-terminal m-GFP fusion from *OPI1* chromosomal locus were grown into log phase in defined media containing 0.2 mM or 5 mM inositol. Cells were imaged by confocal microscopy. Bar represents 5 μm. *Left panels* show GFP channel and *right panels* show DIC image superimposed to GFP signal. m-GFP, monomeric-GFP; LOF, loss of function; LZ, leucine zipper.

To gain insight about the role of the LZ into Opi1 localization and function, Opi1 was tagged at its C terminus with monomeric-GFP. Both Opi1^WT^-mGFP and Opi1^N150D^-mGFP expressing strains grew indistinguishable from their untagged counterparts (Fig. S4). We exploited the synthetic interaction between inositol deprivation and further ER stress upon exposure to the protein glycosylation inhibitor tunicamycin and suboptimal temperatures to score growth differences between *OPI1* alleles. Considering the phenotypic spectrum associated with mutations in the LZ, LOF LZ variants Opi1^C142Y^ and Opi1^L160A^ were also studied. We found that Opi1^L160A^ is hypomorphic and successive point mutations affecting preceding *d* leucine residues 153 and 146 rendered variants comparable to the null allele (Fig. S5). For Opi1 localization, observations were performed on haploid strains expressing Opi1-mGFP variants from the *OPI1* chromosomal locus. We constructed Opi1-mGFP ORFs carrying the indicated LZ mutations and flanked by the promoter and terminator regions of *OPI1* gene. They were used to “heal” the double strand break introduced by CRISPR/Cas9 in the gene marker of an *opi1*Δ::Kan null strain. Growth plate assays of these haploid strains confirmed the phenotypic spectrum associated with mutations in the LZ (Fig. S6). The expression level of Opi1^N150D^-mGFP was reduced compared to Opi1^WT^-mGFP, whereas Opi1^L160A^-mGFP and Opi1^C142Y^-mGFP abundance were higher than the WT, consistent with Opi1 self-regulation (Fig. S7). This observation rules out that the repressing character of Opi1^N150D^ variant was due to increased Opi1 polypeptide concentration. The level of Opi1^3L^-mGFP variant (having the 3 *d* leucine residues 146, 153, and 160 mutated) was similar to Opi1^WT^-mGFP. Confocal microscopy examination showed that all the variants were nucleoplasmic for inositol-fed cells (Fig. 4*A*). After 2 h of inositol withdrawal, Opi1^WT^-mGFP acquired its typical perinuclear and cortical ER localization whereas Opi1^N150D^-mGFP as well as the LOF variants remained nucleoplasmic. For the LOF variants, it is conceivable that upon the onset of the *opi* phenotype by derepressed *INO1* transcription, the consequent increase of inositol and PI biosyntheses leads to a reduction of PA pools, Opi1 membrane detachment, and nucleoplasmic localization. Aiming to dissociate Opi1 localization from the phenotype of the mutants, we re-assessed the LZ variants in haploids cells carrying a KO allele of the *INO1* gene. Growth plate assays showed that *ino1*Δ cells performed like WT cells provided 5 μM inositol was present in the media (Fig. S8). As observed for the *INO1* parental strains, all the variants were nucleoplasmic when cells were grown on 0.2 mM inositol-containing media (Fig. 4*B*). When *ino1*Δ cells were shifted into 5 μM inositol-containing media, Opi1^WT^-mGFP acquired perinuclear distribution after 6 h of cultivation whereas Opi1^N150D^-mGFP remained nucleoplasmic. In contrast with the observation for the *INO1* parental strains, the three LOF variants acquired perinuclear localization when *ino1*Δ cells were grown on low inositol media for at least 6 h. The relocalization of the LOF LZ alleles indicates that the functionality of the LZ is not essential for Opi1 membrane association but necessary for repression upon membrane detachment. The persistent nucleoplasmic distribution of Opi1^N150D^-mGFP supports the notion that the inability to dimerize by this variant locks Opi1 in a detached and repressive form.

We scored growth phenotypes in diploid cells expressing Opi1 LZ alleles. When assayed in hemizygosis, the variants Opi1^C142Y^, Opi1^L160A^, and Opi1^3L^ surpassed the hemizygous WT and performed like the null allele under inositol limitation and upon further stress by the addition of tunicamycin (Fig. S9). Their recessive character was manifested in heterozygosis both with the WT or with the allele encoding Opi1^N150D^ variant. The hemizygous strain expressing Opi1^N150D^ was equally impaired as those Opi1^N150D^ heterozygous strains expressing the WT or the LZ LOF variants Opi1^C142Y^, Opi1^L160A^, and Opi1^3L^ from the second locus.

The genetic analyses attests to the dominant character of the Opi1^N150D^ variant, whereas the intragenic epistasis data suggests that upon monomerization, other intramolecular events involving the UCR and AID domains are necessary for Opi1 to acquire repressing activity. The phenotypic spectrum of the LZ variants supports a model whereby LZ-mediated dimerization competes the LZ away from its monomeric role into Opi1 repression.

**Figure 5:**
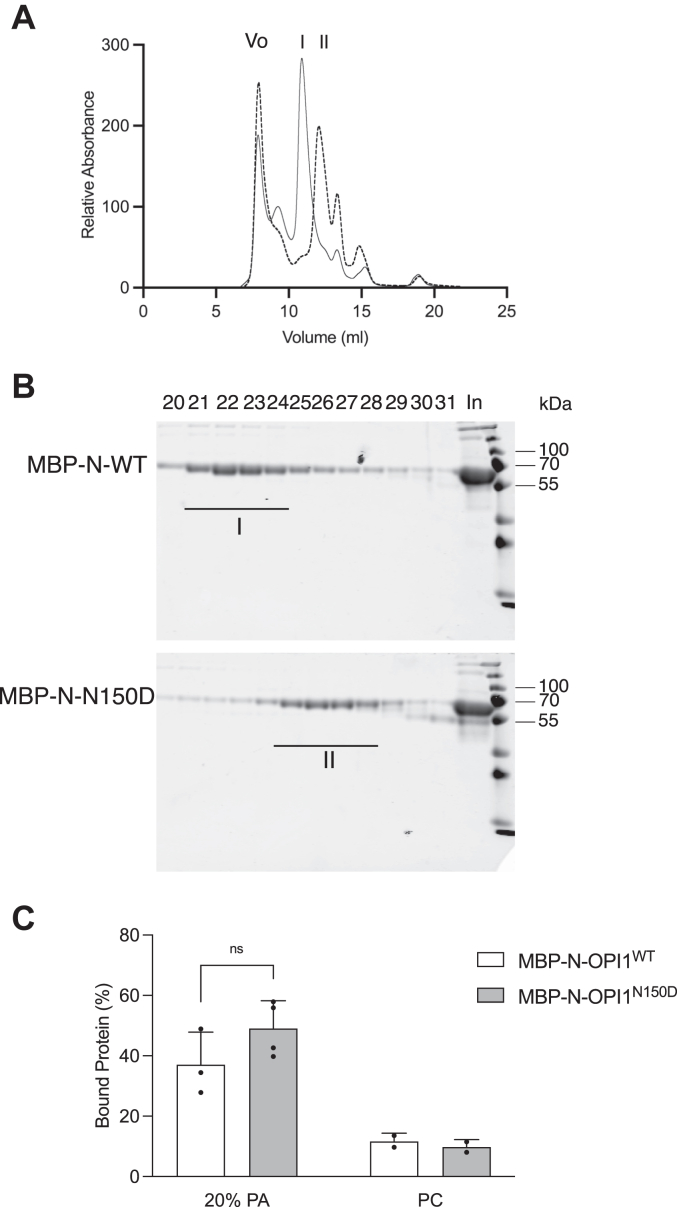
**Association status of MBP-N-Opi1 does not affect its PA-binding activity.***A*, chromatographic profile of 0.5 mg of affinity-purified preparations of MBP-N-Opi1^WT^ (*solid line*) and MBP-N-Opi1^N150D^ (*dashed line*) fractionated by size-exclusion chromatography (SEC) on a Superdex 200 Increase 10/300 Gl column. V_o_ indicates the column void volume (8.4 ml). Peak I corresponds to fraction 22 and peak II corresponds to fraction 26 as indicated in the next panel. *B*, electrophoretic analysis of the chromatographic peaks I and II. Chromatographic fractions of around 0.25 ml were collected and aliquots were analyzed by SDS-PAGE. Fractions numbers are indicated. An aliquot of each affinity-purified preparation were included (In) in each electrophoretogram. *C*, binding activity for SEC-purified MBP-N-Opi1^WT^ and MBP-N-Opi1^N150D^ was assayed towards 100% dioleoyl (DO)-PC or 20% DOPA 80% DOPC liposomes extruded through 100 nm diameter pore filters. Bound probe was fractionated by liposome flotation upon discontinuous sucrose gradient centrifugation. The distribution of the probe along the density gradient was estimated by SDS-PAGE and protein staining. The bound fraction represents the relative amount of probe present in the *top* layer of the gradient. The data represent the average and SD from four independent determinations and is representative of two independent SEC preparations of MBP-N-Opi1^WT^ and MBP-N-Opi1^N150D^. MBP, maltose-binding protein; PA, phosphatidic acid; PC, phosphatidylcholine.

To directly examine the role of the N150D amino acid change within the LZ dimerization interface on the capacity of Opi1 to dimerize, we analyzed by size-exclusion chromatography (SEC) affinity purified preparation of maltose-binding protein (MBP) fused to the N-terminal Opi1 ^1-190^ polypeptide (MBP-N-Opi1) (Fig. S10, *A* and *C*) carrying the WT or the N150D variant (Fig. 5, *A* and *B*). The elution profiles for MBP-N-Opi1^WT^ *versus* MBP-N-Opi1^N150D^ were distinct. Both affinity preparations rendered high molecular weight aggregates eluting at the void volume of the column. The earlier elution of MBP-N-Opi1^WT^ (peak I) compared to MBP-N-Opi1^N150D^ (peak II) implies a larger complex with estimated molecular weights of 190 kDa for peak I *versus* 100 kDa for peak II. Electrophoretic analyses of the eluates from the SEC showed that a polypeptide of the expected molecular weight of 65 kDa for the MBP-N-Opi1^WT^ and MBP-N-Opi1^N150D^ polypeptides was the main constituent of both peaks (Fig. 5*B*). The chromatographic analyses are consistent with MBP-N-Opi1 forming a higher order structure, likely a dimer *via* the LZ, and support the notion that an ionic residue at the LZ position *a* 150 of Opi1 prevents dimerization.

As the LZ is C-terminally adjacent to the PA-binding motif of Opi1 (Fig. 1*A*), it is conceivable that changes within the LZ of Opi1 might affect binding to PA. We assessed binding for SEC-purified MBP-N-Opi1^WT^ and MBP-N-Opi1^N150D^ to PA-containing liposomes. MBP-N-Opi1^N150D^ binding to PA-containing liposomes was comparable to MBP-N-Opi1^WT^, whereas the binding to PC-only liposomes was negligible for both (Fig. 5*C*). This observation suggests that the strong phenotype elicited by Opi1^N150D^ variant is not due to a severe defect in PA binding and is consistent with the observation that the LOF LZ variants are sensitive to inositol-driven membrane changes.

### Dual role of the LZ for Opi1 function

**Figure 6:**
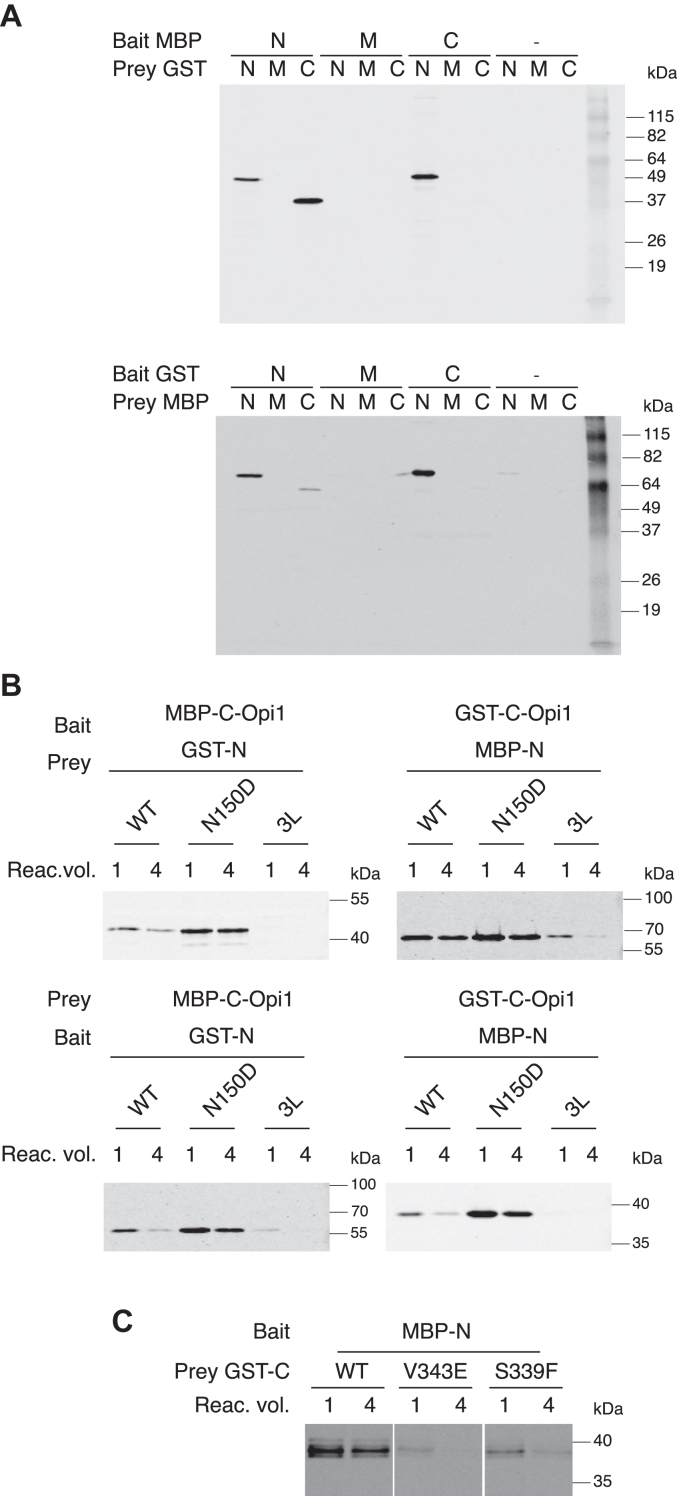
***In vitro* Opi1 interdomain interaction pull-down analyses.***A*, pull-down assays were conducted using affinity-purified N-tagged WT chimeras for the N terminus of Opi1 (N-Opi1, from aa1 to aa190), the middle fragment (M-Opi1, from aa191 to aa290), and the C terminus (C-Opi1, from aa291 to aa404) as prey or bait as indicated. Pull-downed prey was detected by Western blotting. *B*, assays were conducted analogously using WT, N150D, and 3L variants of N-Opi1 and WT C-Opi1 as prey or bait as indicated. The relative volume of the binding reaction is indicated. *C*, GST-C-Opi1 probes carrying mutations S339F and V343E were assayed for their ability to interact to MBP-N-Opi1. The relative volume of the binding reaction is indicated. The data is representative of at least two independent experiments. GST, glutathione-S-transferase; MBP, maltose-binding protein.

To explore Opi1 domain interactions, we performed *in vitro* pull-down assays using recombinant tagged peptides. The Opi1 polypeptide was dissected into three regions containing each conserved domain (Fig. S10*A*): N-Opi1^1-190^, M-Opi1^191-290^, and C-Opi1^291-404^. They were subcloned C-terminally of MBP and of glutathione-S-transferase (GST), and the recombinant chimeras were expressed in bacteria and affinity purified (Fig. S10*B*). Pull-down assays were conducted as follows: the prey was presented to the bait carrying the alternate tag and bound to its affinity resin. After washing the beads and upon specific elution of the bait from its resin, the presence of the prey in the eluate was assessed by Western blotting. The N-Opi1 fusion proteins interacted with each other consistent with the LZ-driving Opi1 dimerization. In addition to self-association, the N-Opi1 chimeras interacted with C-Opi1 chimeras (Fig. 6*A*). The chimeras MBP and GST-M-Opi1 carrying the UCR did not interact with themselves or with N-Opi1 or C-Opi1. Thus, regardless of the tag and regardless of their presence as a bait or as a prey, the known self-association of N-Opi1 was observed in all combinations. Interestingly, we also observed N-Opi1 interacting with C-Opi1 under all conditions.

We next examined if N150D and 3L mutations affected the interaction between N-Opi1 and C-Opi1. These LZ variants were expressed as MBP or GST-N-Opi1 fusions, affinity purified (Fig. S10*C*) and their binding towards GST or MBP-C-Opi1^WT^, respectively, were assessed by pull-down assays. To challenge the specificity of these interactions, binding reactions were carried out at decreasing reactant concentrations. Irrespective of the configuration of the pull-down assay, the 3L mutation attenuated the interaction whereas the binding of N-Opi1^N150D^ to C-Opi1^WT^ was comparable to N-Opi1^WT^ (Fig. 6*B*). We also assessed the impact of loss of function mutations S339F and V343E (Fig. S10*D*) on the interaction of C-Opi1 to N-Opi1^WT^ by pull-down assays. The fraction of GST-C-Opi1^S339F^ and of GST-C-Opi1^V343E^ that remained bound to MBP-N-Opi1^WT^ was reduced compared to GST-C-Opi1^WT^ (Fig. 6*C*). Altogether, the *in vitro* pull-down data is consistent with the notion that beyond Opi1 LZ self-association, a second LZ interaction with the Opi1 AID occurs.

### Nte1 promotes derepression-enhancing Opi1 membrane binding

**Figure 7:**
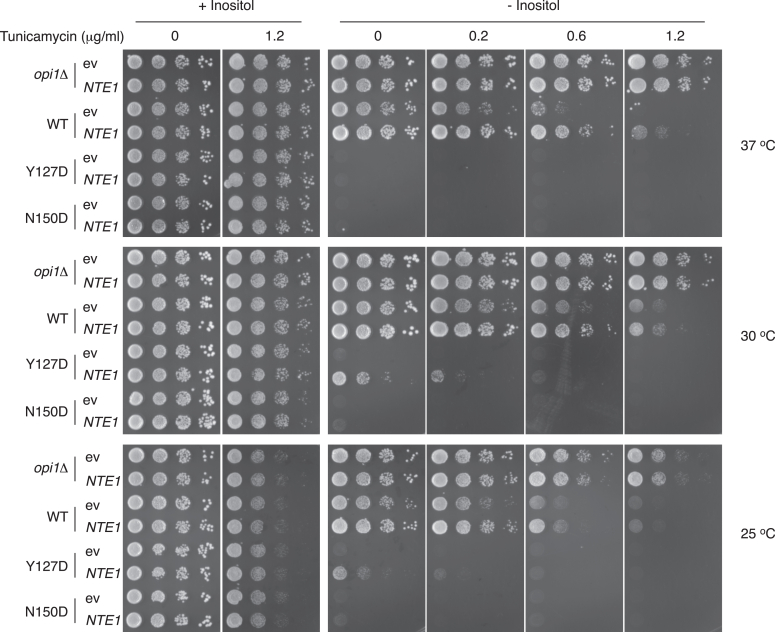
**Functional interaction between PA binding–deficient variant Opi1**^**Y127D**^**and increased *NTE1* gene dosage.** Haploids cells expressing the indicated Opi1 variants from *OPI1* chromosomal locus and transformed with a pRS416 vector carrying or not the *NTE1* gene were grown in synthetic selective media containing 0.2 mM inositol for 16 h at 30 °C. Cells were thoroughly washed and spotted as a 10-fold serial dilution at an initial cell density of A_660 nm_ of 0.4 onto synthetic selective solid media containing or not 0.2 mM inositol and the indicated amount of tunicamycin. Plates were incubated at the indicated temperatures for 3 days. ev, empty vector; PA, phosphatidic acid.

The reversible phenotype of Opi1^L143S^, Opi1^H144L^, Opi1^H144Y^, and Opi1^K147E^ variants to Nte1-mediated rescue depended on Scs2/Scs22 (Fig. 2), suggesting that membrane-binding events cooperate to facilitate LZ dimerization and to oppose repression. To further explore the interaction of increased *NTE1* gene dosage over Opi1 membrane-binding function, the growth phenotype of a haploid strain expressing Opi1^Y127D^, a variant of Opi1 known to have diminished PA-binding activity (14), was examined. Increased *NTE1* gene dose attenuated the *ino* phenotype imparted by the Opi1^Y127D^ variant when inositol-independent growth was assayed under standard conditions (30 °C) (Fig. 7). However, upon further stress by exposure to suboptimal temperatures and to tunicamycin, increased *NTE1* gene dosage did not prevent the phenotype of Opi1^Y127D^-expressing cells. These observations suggest that Nte1-mediated rescue depends on Opi1 membrane-binding events. It is worth to highlight the growth impairment conferred by Opi1^WT^ under higher ER stress compared to the null allele and the ability of Nte1 to attenuate Opi1 repression, implying that even under severe derepressing conditions, a population of Opi1 molecules prevents higher transcriptional activity.

### Nte1 promotes PC saturation

**Figure 8:**
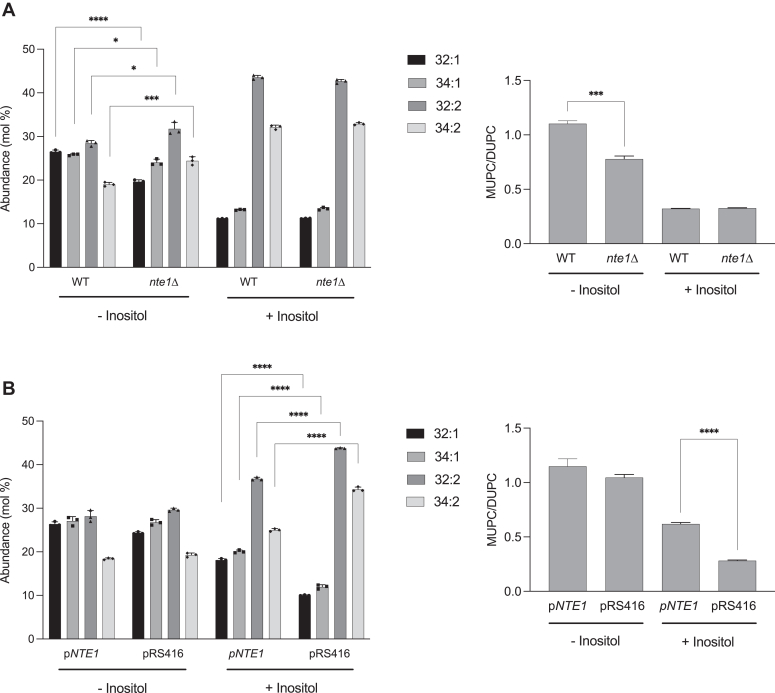
**Nte1 promotes PC saturation.***A*, WT and *nte1*Δ cells were grown in synthetic media containing or not 0.2 mM inositol into log phase at 30 °C. Cells were harvested and lipids extracted. PC species were analyzed and identified by LC/MS-MS. The data represents averages of three independent cultures ± SD. Significant variation of PC species abundance was observed for cells growing without inositol and established by unpaired *t* tests as follows: 32:1 (∗∗∗∗*p* < 0.0001); 34:1 (∗*p* = 0.0156); 32:2 (∗*p* = 0.0239), and 34:2 (∗∗∗∗*p* = 0.0009). *B*, WT cells transformed with a pRS416 vector carrying or not *NTE1* gene were grown in synthetic selective media containing or not 0.2 mM inositol into log phase at 30 °C. Cells were processed for PC species distribution analysis. The data represents averages of three independent cultures ± SD. Significant variation of PC species abundance was observed for cells growing in the presence of inositol and established by unpaired *t* tests for PC species 32:1, 34:1, 32:2, and 34:2 (∗∗∗∗*p* < 0.0001). The *right panel* of each chart represents the ratio of total monounsaturated (32:1 + 34:1) to total di-unsaturated (32:2 + 34:2) PC species. Unpaired *t* tests were performed, and significance was established at *p* = 0.025 (∗∗∗) and *p* = 0.0003 (∗∗∗∗). PC, phosphatidylcholine.

Aiming to gain more insight into the ability of Nte1 to overcome Opi1 repression, we examined its contribution towards PC homeostasis. Importantly, inositol availability is known to (i) impact the PC fatty acyl species distribution profile and (ii) drive flux through the Kennedy pathway for PC synthesis and its turnover by Nte1 (33, 48, 49). In yeast, most PC molecules normally have the acyl composition of either one saturated and one monounsaturated fatty acid (32:1, 34:1 – monounsaturated (MUPC)) or two monounsaturated fatty acids (32:2, 34:2 – di-monounsaturated (DUPC)). This difference can easily be expressed as the MUPC/DUPC ratio. PC is enriched in DUPC species when cells are grown with inositol whereas a more saturated profile is observed for inositol-starved cells (48). PC remodeling through GPC re-acylation into a more saturated PC profile takes place upon inositol scarcity (49). The extent of PC turnover mediated by Nte1 (33, 48) and the induction of the GPC acyltransferase Gpc1, responsible for GPC re-acylation into a more saturated species profile (49), posit these two enzymes in a pathway for adjusting PC species profile to inositol deprivation. Both *nte1*Δ and *gpc1*Δ cells exhibited a less saturated PC profile under those conditions (48, 49). We looked at the distribution of the most abundant PC species, 32:1; 34:1; 32:2, and 34:2, by LC-MS/MS analysis. For cells grown with inositol, the PC species profile was indistinguishable between WT and *nte1*Δ cells with both having a MUPC/DUPC ratio of 0.3 (Fig. 8*A*). Under inositol starvation, the PC from WT cells exhibited a more saturated fatty acid profile, whereas in *nte1*Δ cells, there was a less saturated PC profile resulting in a reduction of the MUPC/DUPC ratio from 1.1 to 0.8, confirming a previous observation (48). We next assessed the impact of *NTE1* gene dosage on PC species distribution profile in the absence and the presence of inositol. The already saturated profile for inositol-starved cells was unaffected by Nte1 overexpression; however, in the presence of inositol, the MUPC/DUPC ratio was 0.6 for cells carrying *NTE1* on a low copy vector *versus* 0.3 for empty vector control cells (Fig. 8*B*). Overall, the data are consistent with Nte1 preferentially hydrolyzing DUPC species. One prediction from this data would be Nte1 activity increases the unsaturated fraction of the cellular pool of FFA.

**Figure 9:**
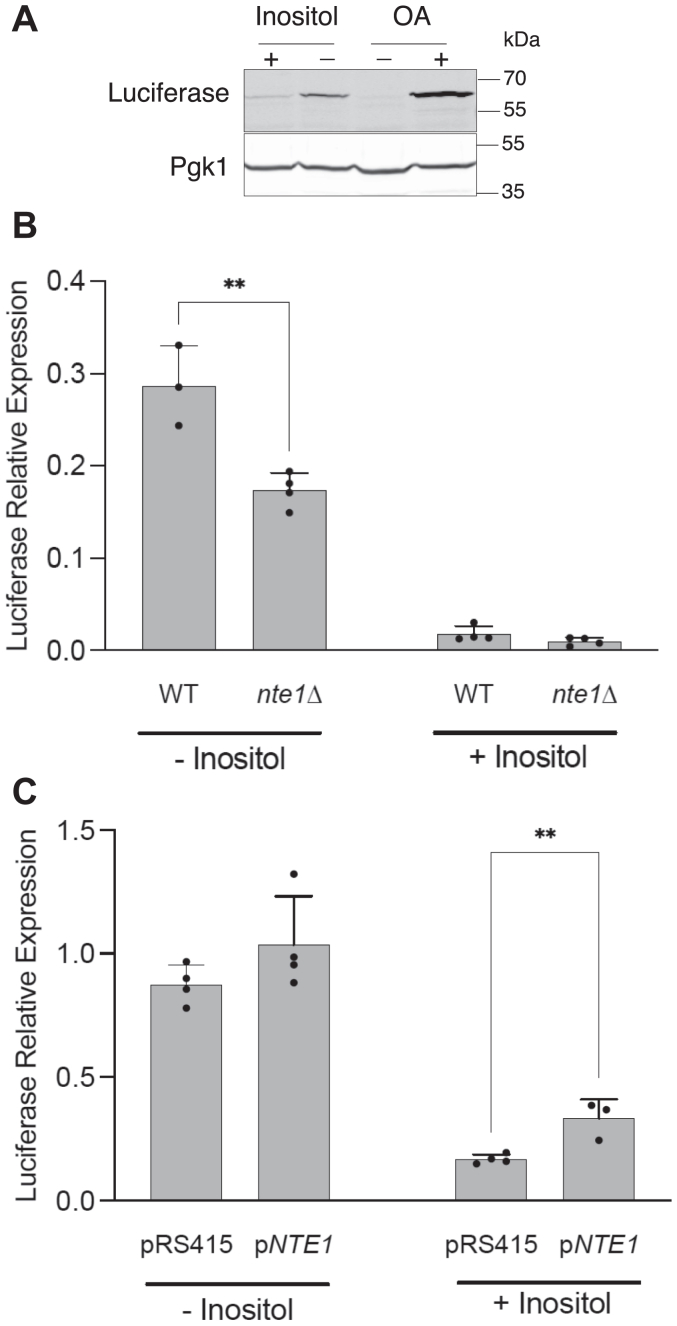
**Inositol deprivation activates Oaf1 signaling.***A*, WT cells transformed with a plasmid carrying a firefly luciferase gene reporter for Oaf1-dependent transcriptional response driven from the *FOX3* promoter were grown into log phase in the presence or the absence of 0.2 mM inositol. Whole cell extracts were prepared and analyzed by SDS-PAGE. Luciferase was detected by Western blotting and Pgk1 was assayed as a loading control. Whole cell extract from cells grown in oleic acid (OA) containing media was included as positive control. *B*, Western blot analysis of inositol-dependent expression of Oaf1 reporter for WT and *nte1*Δ cells. Four fresh transformants of each genotype carrying the Oaf1 reporter gene on a plasmid were grown overnight on selective define media containing 0.2 mM inositol. Cells were thoroughly washed and inoculated into the same media containing or not 0.2 mM inositol at an initial A_660nm_ 0.2. Cells were grown into log phase (A_660nm_ > 0.8), harvested, and processed for Western blotting. Unpaired *t* test was performed, and significance was established at *p* = 0.0051 (∗∗). *C*, effect of increased *NTE1* gene dosage on the expression of Oaf1 luciferase gene reporter. Four fresh transformants with an empty vector or carrying a copy of *NTE1* gene were grown into log phase on selective define media containing or not 0.2 mM inositol. Cells were harvested and processed for firefly luciferase detection by Western blotting. Unpaired *t* test was performed, and significance was established at *p* = 0.0082 (∗∗).

To explore if unsaturated FFA level in the cell is modulated by inositol availability and if Nte1 contributes to this regulation, we took advantage of the oleic acid (OA, 18:1)-activated transcription factor Oaf1. Oaf1 directly binds OA and regulates the expression of genes involved in fatty acid β-oxidation (50, 51, 52). We used a previously characterized reporter where the Oaf1 transcriptional activation sequence drives expression of firefly luciferase. An increase in OA level is known to increase activity of this reporter (50). We observed for inositol-starved cells a higher level of expression of the luciferase reporter than inositol-fed cells (Fig. 9, *A* and *B*). The absence of Nte1 led to a significant decrease on the expression of the reporter when inositol-starved cells were examined, whereas increasing *NTE1* gene dosage elevated luciferase expression for inositol-fed cells (Fig. 9, *B* and *C*). The differential activation of this reporter upon Nte1 variation reveals the contribution of Nte1 to the onset of this response and is consistent with Nte1 preferentially increasing the cellular level of free OA and simultaneously decreasing the unsaturated fatty acid content of PC.

## Discussion

**Figure 10:**
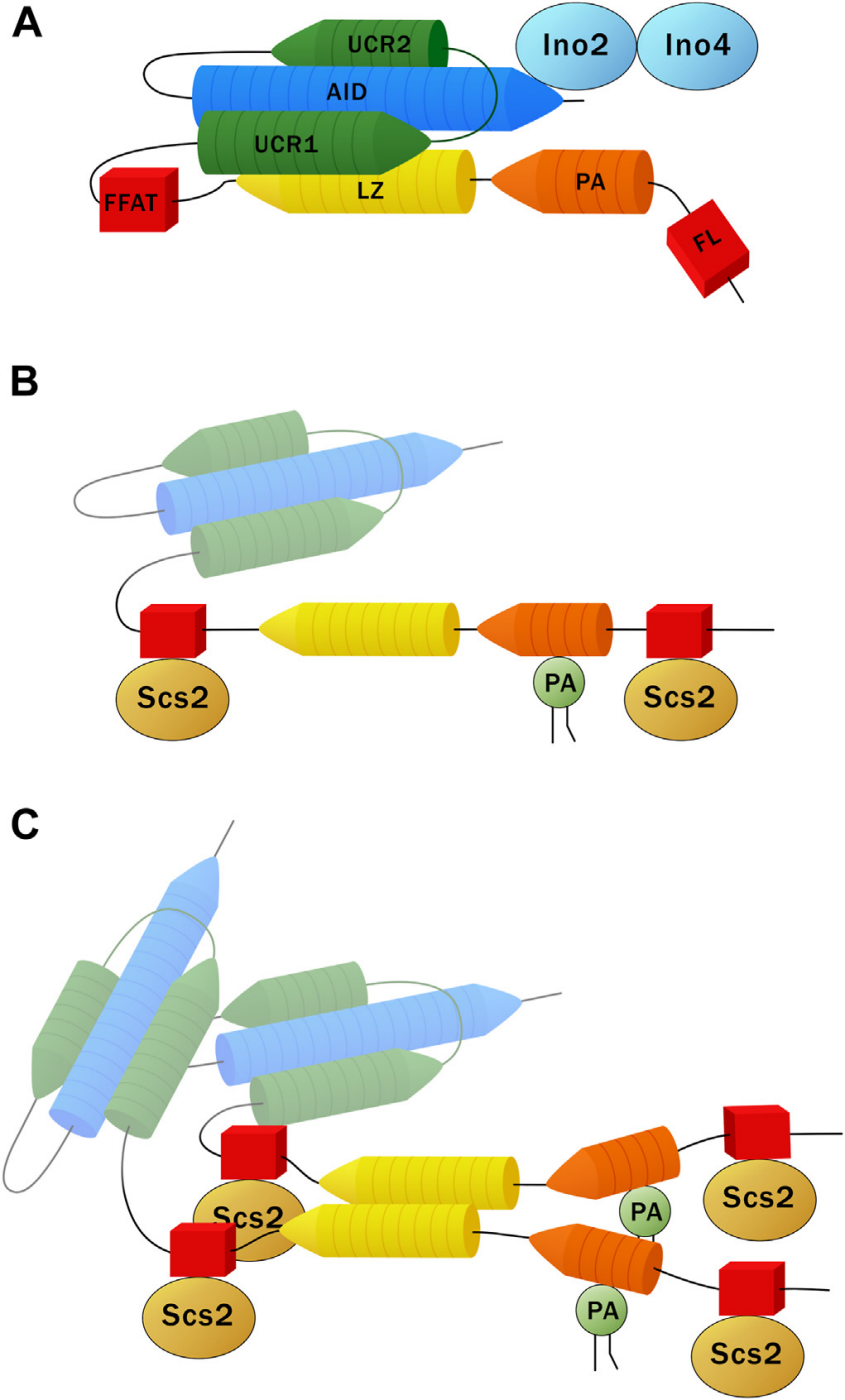
**LZ-mediated molecular transitions underlie Opi1 signal transduction mechanism.***A*, schematic representation of Opi1 monomer structure based on the conserved fold of an anti-parallel four-helix bundle predicted by Alphafold for the Opi1 protein class. The core formed by the LZ (*yellow*) and the AID (*blue*) is connected by a two-helix hinge, UCR1 and UCR1 (*green*), completing the bundle. The PA-binding region and the FFAT and FL motifs are indicated in *orange* and *red*, respectively. This monomeric form interacts with Ino2 and prevents transcription from UAS_INO_-driven genes. *B*, conformational transition from this tight-folded form into an “open” one, whereby the LZ is dislodged away from the AID, is favored upon membrane binding events depending on the PA-binding region and the FFAT and FL motifs. *C*, focal enrichment of monomeric Opi1 over PA/Scs2 membrane patches promotes LZ dimerization stabilizing a membrane-bound nonrepressive form. The inability of Opi1^N150D^ to undergo dimerization locks the variant into a detached and repressive form. Declining level of PA attenuates Opi1 membrane binding, favoring LZ-LZ dissociation, re-acquisition of monomeric LZ-AID interaction, and repression over Ino2/Ino4-driven promoters. The ability of Opi1 to undergo LZ-mediated transitions might also affect its nuclear export: LZ LOF variants as well as Opi1^N150D^ accumulate in the nucleus, whereas Opi1^WT^ acquires some tubular and cortical ER distribution upon inositol withdrawal. Our model for Opi1 signal transduction whereby the LZ quenches its hydrophobic moment either interacting intramolecularly to the AID or through homodimerization does not account for the roles of AID and UCR into a dimer membrane-bound nonrepressing Opi1 form. These domains are represented as “ghosted” regions for membrane-bound Opi1. Highly divergent regions of *Saccharomyces cerevisiae* Opi1 like its N terminus containing Opi1-Sin3 Interacting Domain (OSID; aa. 45–106) or its polyglutamine stretch spanning from Q286 to Q310 were omitted for simplicity from the schematic representations of Opi1 structure. AID, activator interacting domain; ER, endoplasmic reticulum; FL, FFAT-Like; LOF, loss of function; LZ, leucine zipper; PA, phosphatidic acid; UCR, uncharacterized conserved region.

The attributes of Opi1, and the Opi1 variants reported here, are coherent with the LZ of Opi1 being a major regulator of Opi1 structure and function (Fig. 10). The LZ of Opi1 appears to enable Opi1 dimerization, with dimerization decreasing the capacity of Opi1 to repress transcription of PL biosynthetic genes. Opi1 LZ exhibits canonical features known to allow reversible parallel homodimerization. It is a short helix of three heptad repeats forming a hydrophobic face of two rows of aliphatic amino acids: leucine residues at the *d* positions of the heptads (L139, L146, L153, and L160) and L143, I157, and V164 at the *a* positions (Fig. 1*A*). The asparagine at the reminder *a* 150 position is thought to dictate specificity by allowing electrostatic bonding across of the otherwise hydrophobic dimerization interface and to modulate affinity by reducing the stability of the dimer. Instead, an ionic residue would prevent LZ homodimerization by charge repulsion. It was shown that the recombinant chimera MBP-Opi1^1-180^ undergoes dimerization in a concentration-dependent manner, suggesting the LZ allows dynamic dimerization (14). The Opi1^N150D^ variant is dominant, and loss of function mutations associated with the other two conserved domains prevented repression when were present in *cis*, consistent with monomeric Opi1 as the repressive form.

It is conceivable that upon monomerization, the LZ engages in another interaction. Dismantling the LZ by mutating *d* leucine residues deprives Opi1 of transcription-repressing activity. This observation argues against the notion that mere monomerization confers repression and supports a model whereby dimerization competes a LZ interaction necessary for repression. The observation that Opi1^N150D^ imposes repression at low intracellular concentration is congruent with a model whereby the LZ interacts intramolecularly with another domain for Opi1 to acquire repression. *In vitro* pull-down assays using recombinant full-length proteins showed that loss of function variant Opi1^L160A^ exhibits reduced interaction with Ino2, suggesting that the LZ is necessary for the AID to physically interact with Ino2 (39, 53). Recently, the predicted monomeric structure of Opi1 from *S. cerevisiae* and *C. albicans* were available (54), and more recently, the Alpha Fold database was populated with many other Opi1 homologs structures (55). They share a common fold of a four-helix bundle where an antiparallel core formed by the LZ and the AID is connected by the UCR, which itself folds into a two-helix hinge along the AID helix completing the bundle. Our epistasis analyses show that LOF mutations located in the UCR or the AID attenuated the phenotype imparted by the N150D mutation when were present in *cis*, implying that upon monomerization, other molecular events are necessary for Opi1 to acquire repression. We explored interdomain interactions by dissecting Opi1 into its three conserved domains and performing pull-down assays using recombinant tagged chimeras. We found that a chimera Opi1^1-190^ interacts with a chimera Opi1^291-404^ supporting the notion that LZ–AID interaction is necessary for Opi1 to acquire repression. LOF mutations associated with both domains, 3L in the LZ and S339F and V343E in the AID, attenuated the interaction. Importantly, it was unaffected by the N150D mutation. Disabling the LZ by replacing *d* leucine residues compromises the zipper for any role. Instead, the N150D mutation impedes homodimerization by charge repulsion, but the fold of the LZ remains mostly unaltered and can interact with the AID. The features of Opi1^N150D^ variant reported here satisfy a model whereby LZ dimerization antagonizes monomeric LZ–AID interaction and repression.

The domain array of Opi1 flanking the LZ supports the view that membrane-binding events driven by the PA-binding region or by FFAT and FFAT-like motifs would stabilize a monomer form where the LZ is dislodged away from the interaction to the AID. The similar affinity of MBP-N-Opi1^WT^ and MBP-N-Opi1^N150D^ for PA-containing liposomes as well as the nuclear localization of the LZ LOF variants and their susceptibility to inositol-induced membrane changes imply that LZ integrity is not required for Opi1 membrane association but necessary for repression. The dependence on Scs2/Scs22 for the differential sensitivity of the Opi1^L143S^, Opi1^H144L^, Opi1^H144Y^, and Opi1^K147E^ variants to increased *NTE1* gene dose supports the notion that focal concentration of monomeric Opi1 upon binding to PA/Scs2 membrane patches would favor LZ homodimerization. The synergy of these events opposing monomeric LZ–AID interaction is at the core of the transduction mechanism proposed for the Opi1 protein class. We postulate monomeric LZ–AID interaction as a key molecular event triggering Opi1 repression and as such the subject of sensory and regulatory inputs.

Increased lipid saturation is known to elicit the unfolded protein response (56, 57). Considering that the relative abundance of PC is even higher when inositol is limiting, the remodeling of PC *via* GPC re-acylation into a more saturated profile contributes to generating membrane changes for restoring ER homeostasis upon inositol deprivation. The extensive PC remodeling triggered by inositol withdrawal could affect the levels and species distribution of the FFA pool. We show here that for inositol-starved cells, a gene reporter for Oaf1 transcriptional factor was induced several fold. The increased expression of this reporter upon augmented *NTE1* gene dosage observed for inositol-grown cells parallels the shift into a more saturated PC profile suggesting that Nte1 contribution towards PC remodeling leads to an increase in the unsaturated fraction of the cellular pool of FFA. We were not able to score significant variations for PA levels or a shift for the PC/PE ratio that could account for Nte1 effects on Opi1 function. We speculate that a shift towards unsaturation for the FFA pool could enhance Opi1 membrane binding and derepression.

In summary, we reconcile evidence pointing to the LZ as an essential domain for Opi1 function into a model whereby membrane binding/LZ dimerization antagonizes monomeric Opi1 LZ/AID and transcription repression of lipid synthesis genes. Our proposed model provides a framework for further studying Opi1 biology.

## Experimental procedures

### Strains and culture conditions

Yeast cells were maintained in YEPD (1% yeast extract, 2% bacto-peptone, 2% dextrose) medium or synthetic complete medium (SC: 0.17% yeast nitrogen base without amino acids and without ammonium sulfate, 0.5% ammonium sulfate, 2% dextrose, 0.2% SC mix). Medium was supplemented alternatively as required for plasmid maintenance with SC drop-out mixes. Of note, inositol concentration for SC media is 486 μM, yeast nitrogen base contributing 11 μM, and SC mix contributing 475 μM. Yeast cells were routinely grown at 30 °C in synthetic defined medium (0.67% yeast nitrogen base without inositol and without amino acids) supplemented as required for plasmid maintenance and nutritional auxotrophies and with the indicated amount of inositol.

For the characterization of *OPI1* alleles, a haploid *opi1*Δ BY 4742 was generated by sporulation and selection of a heterozygous diploid *OPI1*/*opi1*Δ::KanMX4 BY4743 obtained from Euroscarf. The screen strain BY4742 *opi1*Δ::HYG *scs2*Δ::NAT *scs22*Δ::KanMX4 was generated by standard genetic crosses of single mutants obtained from a BY4741 deletion collection. The genetic KanMX4 marker was swapped into nourseothricin acetyltransferase or hygromycin resistance markers using plasmid pAG25 or pAG32 as described (58). Other strains used in this study are listed in Table S1.

### Plasmid construction

Plasmid pPM170 carries both upstream and downstream regions of *OPI1* ORF cloned contiguous and jointed through an *Xma*I site. A DNA fragment extending from nt −274 to +3 of *OPI1* gene (numbered from start codon) was amplified with primers 5′-U-OPI1-*Sal*I and 5′-D-OPI1-*Xma*I and positionally cloned into pRS415 vector using those restriction enzymes. A second DNA fragment from nt 1209 to 1597 of the *OPI1* gene including *OPI1* stop codon (nt 1213–15) was generated by PCR using primers 3′-U-OPI1-*Xma*I and 3′-D-OPI1-*Sac*I and subcloned contiguous of the 5′ end of *OPI1* into the pRS415 backbone using *Xma*I and *Sac*I to generate pPM170.

The pPM170 plasmid was linearized by *Xma*I/*Nsi*I double digestion for subcloning the mutagenized *OPI1* PCR amplicon by *in vivo* plasmid gap repair. At both ends of the mutagenized *OPI1* fragments, there is ∼0.1 Kb overlapping with the 5′ and 3′ flanking regions of *OPI1* gene present in the pPM170 plasmid. The WT allele of the *OPI1* gene was cloned by PCR amplification from genomic DNA using primers 5′-U-*OPI1*-*Sal*I and 3′-D-*OPI1*-*Sac*I and positional subcloned into vector pPM170 by inserting a 1.7 Kb *Nde*I/*Xba*I fragment to render plasmid pPM169. Primers used in this work are listed in Table S2.

The *OPI1* alleles encoding the variants C142Y, L143S, H144Y, K147E, and L160A were subcloned from their original plasmid source into plasmid pPM169 by replacing the 0.6 Kb *Aat*II/*Bgl*II DNA fragment. The allelic variants H144L and N150D were introduced by site-directed mutagenesis into pPM169. The variants L153S L160A and L146A L153S L160A (3L) were constructed by sequential site-directed mutagenesis of the allele encoding the L160A variant carried on a pRS415 plasmid. The alleles encoding the variants K226E, L255S, S339F, and V343E were cloned from genomic DNA by PCR amplification followed by swapping a 0.45 Kb fragment generated by *Bgl*II and *SnaB*I double restriction digestion into pPM169 backbone. The allelic variant L252F was subcloned from its original plasmid source by *Bgl*II/*SnaB*I 0.45 Kb fragment swapping. All the *cis* constructions carrying the LOF mutations located downstream of the *Bgl*II site were built by 0.45 Kb *Bgl*II/*SnaB*I fragment swapping into a pRS415-OPI1^N150D^ backbone. For the construction of the double N150D K226E mutant, the N150D mutation was introduced by site-directed mutagenesis into the TOPO clone carrying the mutation K226E for subsequent subcloning of the 0.6 Kb *Aat*II/*Bgl*II DNA fragment into pPM169. Plasmids used in this study are listed in Table S3.

### Screen for repressing *OPI1* mutants

Mutagenic PCR reactions were performed in 25 μl containing 10 ng of yeast genomic DNA, 1x *Taq* polymerase reaction buffer, 5 pmol of 5′-*OPI1-*M primer, 5 pmol 3′-*OPI1-*M primer, and 2 units of *Taq* DNA polymerase. Mutagenic conditions were achieved by varying deoxynucleotide concentrations (dGTP, dTTP, and dCTP 0.2 mM, dATP 1 mM) and increasing divalent cation concentration (4.5 mM MgCl_2_ and 0.5 mM MnCl_2_). Several pools of amplicons generated by error-prone amplification of *OPI1* gene were subcloned by gap repair into linearized pPM170. Briefly, the gapped plasmid plus the PCR-amplified *OPI1* products were cotransformed into an *opi1*Δ *scs2*Δ *scs22*Δ carrying pRS416-*NTE1* plasmid. For gap repair, 120 ng of PCR product was cotransformed with 60 ng of *XmaI*/*NsiI* double-digested pPM170 plasmid into 2.5 × 10^7^ cells. Transformants were selected in SC media minus uracil and leucine. Typically, 4000∼6000 colonies were obtained from a standard transformation reaction. Colonies were replicated onto synthetic define selective media lacking inositol at 37 °C and those performing poorly under those conditions were further analyzed.

### Opi1-mGFP fusion construction and chromosomal integration into the *OPI1* locus

To generate the Opi1-mGFP fusions for the studied *OPI1* alleles, a 1.3 Kb DNA fragment containing the 3′ end of *OPI1* ORF fused to GFP-encoding sequence was amplified from genomic DNA of a strain encoding *OPI1*-GFP::*HIS3* with primers F2-G and 3′-GFP-*HIS3*-*Sac*I. The mutation A206K preventing GFP dimerization was introduced by site-directed mutagenesis. This fragment was subcloned downstream of the *OPI1* alleles carried on a pRS415 vector by replacing the *SnaB*I/*Sac*I fragment to render Opi1-mGFP ORF under the control of *OPI1* promoter. A 0.4 Kb DNA fragment containing the 3′ region downstream of *OPI1* ORF was PCR amplified from genomic DNA using primers *Asc*I-3′-Up-*OPI1* and 3′-D-*OPI1*-*Sac*I. This fragment was subcloned downstream of *OPI1*-mGFP ORF, carried on a pRS415 backbone, by restriction digestion and ligation at the *Asc*I and *Sac*I sites. The resulting plasmids carrying *OPI1*-mGFP variants flanked by the upstream and downstream sequences of *OPI1* gene served as donors of healing DNA fragments for a double strand break introduced by Cas9 at the kanMX4 gene of an *opi1*Δ::kanMX4 haploid strain. The healing fragment was generated by *Xho*I digestion and isolation of the 2.5 Kb DNA fragment. Haploid *opi1*Δ::kanMX4 cells were transformed with the healing DNA fragment and with plasmid pAR1275. This pRS425-based plasmid expresses Cas9 and encodes a guide RNA that targets kanMX4 for a double strand break. Transformed cells were selected on SC-LEU plates; isolated colonies were propagated under nonselecting conditions for plasmid lost and were assessed for G-418 sensitivity as it was regained upon recombination at the *OPI1* locus. Proper integration of the healing fragment was assessed by PCR using primers 5′-*OPI1*g and 3′-*OPI1*g. Positives clones were characterized phenotypically.

### Confocal microscopy

Log phase cells grown in the indicated media were concentrated by centrifugation at 1000*g* for 5 min. A 3 μl aliquot was mounted on thin 0.9% Sea Kem agarose pad prepared in synthetic define medium containing the indicated inositol concentrations. Images of live cells were captured using a Leica TCS SP8 with LIGHTNING confocal microscope using excitation wavelength of 448 nm at 100x magnification. A 1.7× magnification was further applied to all images by the LAS X software (https://www.leica-microsystems.com/products/microscope-software/p/leica-las-x-ls/). Fields were focused manually using DIC. Exposure times and laser intensities were kept constants for all different yeast strains.

### Whole yeast extract preparation

Flasks containing cultures of around 5 × 10^7^ logarithmic cells were immersed in an ice-water bath and media was made to 10 mM NaN_3_, 10 mM NaF, and 1 mM PMSF. Cultures rested at 4 °C for 5 to 10 min, cells were centrifuged, resuspended in ice-cold 10 mM NaN_3_, 10 mM NaF, and 1 mM PMSF, total number of cells equalized across samples and transferred into a screw-cap plastic vial, centrifuged again, and the cell pellet frozen using liquid nitrogen and stored at −80 °C. Cells were thawed in 250 μl of ice-cold 1X Laemmli sample buffer containing 5% β-mercaptoethanol and 1 mM PMSF, and cold glass beads were added up to the suspension meniscus. Cells were lysed by four cycles of 30 s of bead beating intercalating with 60 s on ice. Cell lysates were recovered and clarified by centrifugation at 21,000*g* for 10 min at 4 °C. Appropriated aliquots were analyzed by SDS-PAGE.

### Serial dilution plate growth assay

Singles colonies were grown overnight at 30 °C in 1.5 ml of the indicated media. Cells were washed twice with ice-cold water, their OD at 660_nm_ determined, and ten-fold serially diluted from an initial A_660nm_ of 0.4 in sterile water. Cell suspensions were pinned down onto solid media and incubated for 2 to 4 days at the indicated temperature.

### Expression of recombinant Opi1 peptides

DNA encoding the N-terminus of Opi1 from aa1 to aa190 (N-Opi1: MW 21,526 pI 5.12), the middle fragment from aa191 to aa290 (M-Opi1: MW 11,344 pI 4.5), and the C terminus from aa291 to aa404 (C-Opi1: MW13,230 pI 8.15) (Fig. S10*A*) were amplified by PCR with the primers listed in Table S2, using WT or mutant *OPI1* DNA templates as indicated. Amplified DNA was subcloned into the pCR 2.1-TOPO vector, DNA inserts were sequenced to ensure fidelity, and subcloned into the pGEX-4T-1 or pMAL-c expression vectors using the restriction enzymes indicated for each pair of primers. Recombinant proteins were expressed in BL21-derived NEB Express cells. Bacterial cells from an overnight culture were centrifuged and reinoculated at A_660nm_ ∼0.15 in fresh LB media. For cells carrying pMAL vectors, LB media was supplemented with 0.2% dextrose. Cells were cultured at 37 °C until an A_660 nm_ ∼0.7 was reached and recombinant protein expression was induced at 37 °C for additional 1.5 h by the addition of 1 mM IPTG. Cultures were chilled on an ice-water bath, pelleted at 4 °C, washed once with ice-cold 20 mM Tris–HCl pH 8, 150 mM NaCl, 1 mM EDTA, 1 mM PMSF, and 1 mM DTT, frozen using liquid nitrogen, and stored at −80 °C.

### Protein pull-down assays

Bacterial pellets from 0.5 L cultures were resuspended in ice-cold 20 ml of 20 mM Tris–HCl pH 8, 150 mM NaCl supplemented to 1 mM PMSF, 3 mM DTT, 30 U/ml Benzonase nuclease, 2 μg/ml pepstatin A, and 1× Complete protease inhibitor cocktail. Cell suspensions were sonicated four times for 30 s with 1 min resting in an ice-water bath each cycle. Crude cell extracts were centrifuged at 4 °C for 30 min at 46,000*g* and supernatants were transferred into new tubes. The supernatants were made 20% glycerol 0.05% Triton X-100 and added to 400 μl of amylose resin or glutathione-Sepharose respectively, previously equilibrated in binding buffer (20 mM Tris–HCl pH 8, 150 mM NaCl, 1 mM EDTA, and 1 mM DTT 20% glycerol 0.05% Triton X-100). The binding proceeded for 1 h at 4 °C on a spinning wheel, and the beads were washed 4 times with 10 ml of ice-cold binding buffer. Half of the washed slurry was kept at 4 °C and the other half was subjected to elution using 10 mM maltose or 10 mM glutathione in binding buffer, accordingly. Aliquots of the eluates were analyzed by SDS-PAGE followed by Coomassie blue staining to estimate recombinant protein enrichment and concentration (Fig. S6).

Around 0.5 μg of bait protein present in 15 μl of drained beads was incubated with 1.5 μg of prey protein in 100 μl of binding buffer containing 50 μg/ml of bovine serum albumin for 2 h at 4 °C on a spinning wheel. Beads were washed four times with 1 ml of binding buffer and the bait protein was eluted from the affinity resin using its specific ligand. The presence of the prey protein in the eluates was assessed by Western blotting using monoclonal antibodies against MBP or against GST.

### Quantitative Western blotting

All primary antibodies used for Western blotting were of mouse monoclonal origin. Antibodies against yeast Pgk1 (cat# 459250, RRID:AB_2532235) and against GST (cat# MA4-004; RRID:AB_10979611) were from Thermo Fisher Scientific. Antibody against MBP was from New England Biolabs (cat# E8032S; RRID:AB_1559730). Antibody against GFP was from Takara (cat# 632380; RRID:AB_10013427). Antibody against firefly luciferase was from Abcam (cat# ab16466; RRID:AB_443388). Routinely, Western blotting images based on mouse monoclonal antibodies recognition were acquired using secondary goat anti-mouse IRDye-antibodies from LiCor Biosciences (680RD: cat# 925-68070, RRID:AB_2651128; 800CW: cat# 926-32210, RRID:AB_621842) by scanning on an Odyssey v3.0 (LiCor) and quantified with Fiji ImageJ (NIH) (https://imagej.net/software/fiji/downloads). Statistical analyses were carried out using Prism software (https://www.graphpad.com/features).

### Liposome flotation assay

Binding activity to PL membranes for the MBP-N-Opi1^WT^ and MBP-N-Opi1^N150D^ chimeras was assessed by their cofractionation with liposomes upon flotation on a density gradient. Briefly, lipids were dried in glass tubes under a stream of nitrogen and desiccated overnight under vacuum. Lipids were rehydrated for 2 h in 20 mM Tris–HCl pH 7.4, 150 mM NaCl with intermittent mixing and then subjected to five cycles of freezing/thawing using liquid nitrogen and 42 °C water bath. Liposomes were extruded 15 times through polycarbonate filters of 800, 200, and 100 nm pore sizes following manufacturer instructions (LiposoFast Basic, Avestin Inc). Binding reaction proceeded at room temperature for 45 min in 150 μl of 20 mM Tris–HCl pH 7.4, 150 mM NaCl containing 1.6 mM liposomes and 0.5 μM of the indicated SEC-purified MBP-peptide probe. At the end of the incubation period, the bound protein fraction was separated by liposome flotation on discontinuous sucrose gradients. Succinctly, 100 μl of 75% sucrose prepared in 20 mM Tris–HCl pH 7.4, 150 mM NaCl was gently mixed with the reaction mixture to render 250 μl of 30% sucrose followed by the successive and careful addition of a layer of 200 μl of 20% sucrose and 50 μl of 0% sucrose in the same buffer. Centrifugation proceeded at 22 °C for 1 h at 85,000 rpm in a TL-100 ultracentrifuge using a TLA 120.1 rotor (Beckman Coulter). Four fractions, two of 100 μl and two of 150 μl, were collected from top to bottom and appropriate aliquots were analyzed by SDS-PAGE, stained with Instant Blue, and thoroughly destained with water. Images were acquired using Odyssey v3.0 (LiCor) and quantified with ImageJ (NIH). Bound fraction represents the abundance of the probe in the top fifth fraction relative to its total amount, distributed along the sucrose gradient. Statistical analyses were performed using Prism software. For the preparation of the affinity-purified protein probes, a frozen bacterial pellet from 1L culture was resuspended in 50 ml 20 mM Tris–HCl pH 7.4, 150 mM NaCl supplemented to 1 mM PMSF, 3 mM DTT, 30 U/ml Benzonase nuclease, 2 μg/ml pepstatin A, and 1× Complete protease inhibitor cocktail. Cell suspensions were sonicated four times for 30 s with 1 min resting in an ice-water bath each cycle. Crude cell extracts were centrifuged at 4 °C for 30 min at 46,000*g*. Supernatants were transferred into new screw-cup tubes containing 1 ml of amylose-resin equilibrated in 20 mM Tris–HCl pH 7.4, 150 mM NaCl, and affinity binding proceeded for 1 h at 4 °C with gentle rotation. Amylose beads were washed twice in batch with ice-cold 20 mM Tris–HCl pH 7.4, 150 mM NaCl and transferred into a chromatography column, followed with further washes until protein content neared baseline. MBP fusions were eluted by the addition of 10 mM maltose in 20 mM Tris–HCl pH 7.4, 150 mM NaCl. Fractions of 0.5 ml were collected and those containing the highest MBP-probe enrichment were pooled. MBP-probes content was assessed by SDS/PAGE and its relative abundance estimated. Around 0.5 mg of affinity purified MBP-N-Opi1^WT^ and MBP-N-Opi1^N150D^ were subjected to SEC on a Superdex 200 increase 10/300 Gl column, equilibrated, and developed with 20 mM Tris–HCl pH 7.4, 150 mM NaCl at 0.5 ml/min using an AKTA 25 platform. Fractions of 250 μl were collected after 0.2 column volume was eluted. Molecular weights were estimated based on the calibration of the SEC column using the following markers: Blue-Dextran (Vo), thyroglobulin (670 kDa), β-amylase (200 kDa), alcohol dehydrogenase (150 kDa), bovine serum albumin (67 kDa), carbonic anhydrase (29 kDa), and cytochrome C (12.5 kDa).

### PC species distribution profiling by LC-MS/MS

Yeast cells growing logarithmically were harvested, thoroughly washed with ice-cold H_2_O, and flash frozen in liquid nitrogen. To 5 OD units of cells, 0.5 ml of CHCl_3_:CH_3_OH (1:1) and 10 μl of Splash Lipidomix mass spec standard were added. Cells were broken by glass bead beating and cell lysates transferred into Teflon lid screw glass tubes with the addition of 0.5 ml of CHCl_3_:CH_3_OH (2:1). Extraction proceeded for 30 min at room temperature with vigorous agitation. For lipid partitioning, 0.5 ml of CHCl_3_:CH_3_OH (5:1) and 1.5 ml of H_2_O were added, tubes subjected to vigorous agitation for 30 min at room temperature, and centrifuged at 3000 rpm for 10 min in an Allegra X-12R centrifuge using a swinging bucket SX4750 rotor. Around 0.5 ml of chloroformic bottom layer were removed and dried on a Speed Vac. Dried material was dissolved in 50 μl of lipidomic resuspension buffer (See Buffer composition at Time = 0 in Table S4). Resuspended samples were analyzed by liquid-chromatography electrospray tandem mass spectrometry operated in data-dependent acquisition mode. For this purpose, a Vanquish UHPLC (Thermo Fisher Scientific) system was coupled to a QExactive orbitrap mass spectrometer *via* a heated electrospray ionization source and controlled by XCalibur software 4.0 (Thermo Fisher Scientific) (https://www.thermofisher.com/order/catalog/product/OPTON-30965). Chromatographic separations of the lipid extracts were performed by injecting 10 μl of the lipid extracts on an Accucore C30 column (250 × 2.1 mm I.D., particle size: 2.8 μm, Thermo Fisher Scientific) kept at 30 °C. The mobile phase composition and chromatographic conditions are shown in Table S4. Each lipid extract was successively injected in both positive and negative ion modes. The data acquisition parameters for both methods are described in Table S5. Data analysis was performed using LipidSearch software version 5.0 (https://www.thermofisher.com/ca/en/home/industrial/mass-spectrometry/liquid-chromatography-mass-spectrometry-lc-ms/lc-ms-software/multi-omics-data-analysis/lipid-search-software.html) for lipid identification and quantitation. The data analysis parameters used are detailed in Table S6. The instrument was externally calibrated to 1 ppm using ESI negative and positive CalMix calibration solutions (Thermo Fisher Scientific). Statistical analyses were carried out using Prism software.

### Reagents

*Taq* DNA polymerase, Platinum *Taq* DNA polymerase High Fidelity, and pCR2.1-TOPO vector were from Invitrogen; Sea Kem Gold Agarose and Surfact-Amps X-100 were from Thermo Fisher Scientific; Benzonase was from Sigma; protease inhibitors cocktail Complete was from Roche. QuickChange mutagenesis kit was from Stratagene; amylose-resin, *E. coli* Express cells and restriction enzymes were from New England BioLabs; glutathione-Sepharose 4 Fast Flow was from Cytiva. Yeast nitrogen base without amino acids and without inositol was from Formedium. Synthetic complete drop-out mixes and yeast nitrogen base without amino acids and without ammonium sulfate were from Sunrise Science. Lipids and Splash Lipidomix mass spec standard were from Avanti Polar Lipids. Instant Blue Coomassie staining was from Abcam.

## Data availability

All the data are contained within the manuscript or the supporting information.

## Supporting information

This article contains supporting information.

## Conflict of interest

The authors declare that they have no conflicts of interest with the contents of this article.
