# Supplementary material for: The leucine zipper domain of the transcriptional repressor Opi1 underlies a signal transduction mechanism regulating lipid synthesis
Source: J Biol Chem. 2023 Oct 31;299(12):105417. doi: 10.1016/j.jbc.2023.105417 (PMC10709064; doi:10.1016/j.jbc.2023.105417)
Supplement: Supporting Information [file mmc1.pdf]

**The leucine zipper domain of the transcriptional repressor Opi1 underlies a signal transduction mechanism regulating lipid synthesis**

J. Pedro Fernández-Murray, Mahtab Tavasoli, Jason Williams, and Christopher R. McMaster

Supplemental information included

Supporting Figures S1-S10

Supporting Tables S1-S6

```
S.cerevisiae ----------------------------------M-----SENQRLGLSEEEVEAAEVL 20
C.glabrata   --------------------------MDTRRGWIH-----NYISFSGLSEEDVEAAEAL 28
Y.lipolytica -------------------------------------------MPKALSSKSLDAASIH 16
C.albicans   MTAPSISNQQLPPPPYSKTDNLPTSNYQSSLNGSVQGQDQQVQAQVQGQAPELVSAAETL 60
                                                           . : : :.**.

S.cerevisiae GVLKQSCRQKSQPS---------------------------------------------- 34
C.glabrata   DVLRNSHVDADVEVEEDRSKKRVRDGEDEDGGHVRRRKASVRDRSDSRMSQLSNVSNAST 88
Y.lipolytica -------------MA-------------SNGAPP--------------L------IQKSS 30
C.albicans   TSLT-----------------------RNGTPPSDADTVAMDDAQSPV------I---- 86


S.cerevisiae -EDVS----QADKMPASESSTTPLNILDRVSNKIISNVVTFY--DEINTNKR-P------ 80
C.glabrata   EERVVRVNSHEDSQSKRQEEESLFDKVCRNSNEILTNMGSFF--EEMNSNVF-MEGGSDV 145
Y.lipolytica LHDFERLKSGIDDIQNSDT----------NGTQYSVDVGSQGLRMRIQTQGYAPSGNSNR 80
C.albicans   ---LP----PIHPQHQRHPIVSTVSMV--ARHPIVMNAVKYYETSKRNYPSFNYAAGI-- 135
                .       .     .                  :  .     . :

S.cerevisiae -----------------LKSIGRLLD-----------------DDDDEHDDYD-YNDDEF 105
C.glabrata   PEEHPNGYVRPRRDSRSISTTGADADTESQGTQGSY------NGKWDTWSSRS-YNDRQY 198
Y.lipolytica GS-----PVPPSPALSTSTSSGSTSAAASRSTSSPV------PQQPQTAAT---AAGGPR 126
C.albicans   --------V-ESAAIPVVNNIEAKLNTRHQTRQASAVSSTDITPTNSNFGDYKIQHDGKN 186
                               ..                         .         .
                                                 142Y     150D        160A
S.cerevisiae FTNKRQKLSRAIAKGKDNLKEYKLNMSIESKKRLVTCLHLLKLANKQLSDKISCLQDLVE 165
C.glabrata   -YSKRKALSEALAKGRYNLREYKLTMSIESKKRLITCLHLLKLANKQLSDKVAYLQDAVE 257
Y.lipolytica PGQPRSAWQEVL-----ISATSLASLSQDSRKRLRYCLHLLKLANAHLASTVTKLQGAIA 181
C.albicans   SLQKKRRFSASS-----QTTNISSYSSIDTKKRLQFCINILKLANTNISSKVEFLQEKID 241
               . :   .                * :::***  *:::*****  :::..:   **   :

S.cerevisiae KEQVHPLHKQDGNARTTTGAGEDETSSDEDDDDEEFFDASEQVNASEQSIVVKMEVVGTV 225
C.glabrata   KEQELANGEE---VKKETRALNGEHSGPQNDDDLEFYDASESVDQNSG--DLGLEIVGTV 312
Y.lipolytica EETAYSLAQSIAAN-----------HVP-HNERQAYLHQPPSAEPALSITALKADVVSTI 229
C.albicans   ETEIAVKEEREKLI--------AQ-----KSHDSNS-----TNTTEQATQKTKTEIVGTV 283
             :       :                     ...                       ::*.*:
           226E                             252F 255S
S.cerevisiae KKVYSLISKFTANSLPEPARSQVRESLLNLPTNWFDSVHSTSLPHHASFHYANCEEQKVE 285
C.glabrata   KKVYSLISKYTGSSLPEPARSQVRESLLNLPSNWNTSVHNGFKNNGTGIMTAS------- 365
Y.lipolytica RKIIKVVSQYAGNSLPEPARSHIRTYILGLPSRWASTTASTNITPTRSPAGSQ---SPVG 286
C.albicans   KKIIHLISNFRPSSLGDTSVTN---GLSPVSSNGSQSN--------QDFELKNAIRD--- 329
             :*:   ::*::   .** : : ::     :  : :.   :          .    .
                                                               339F 343E
S.cerevisiae QQQQQQQQQQQQQLLQQQLLQQQQQKRNKDGDDSASPSSSVTANGKVLILAKESLEMVRN 345
C.glabrata   --------------------------SSTDSLSSYSSILPVSSNGKYLILAKESLNMVQS 399
Y.lipolytica SPKECVTPDHQGPPLPTNTVSSPVP-EGPSDEQLSRHRIEVEAGGKVLILANEALDMLGN 345
C.albicans   ----------IIMSLPQSLQQQQQQQSGPPS---------NSQDDVIFKFAKESLVMISK 370
                                        .  .            ..  :  :*:*:*  *:  .

S.cerevisiae VMGVVDSTLGKAEEWVKQKQEVKEMIRERFLQQQQQYRQQQQKDGNYVKPSQDNVDSKD- 404
C.glabrata   VIDVVDSTLGRAEEWVKQKQELKEMIKKKFLEQQEHQKMLGIGTESL-DQNESSVKKEQP 458
Y.lipolytica IISIVDGTLERAEGWCEGINRVKQRVGLGETAP-----------GAAGEPGASTEASAEA 394
C.albicans   LTQIFTEKLDQVEHWVNGDEEQEQKLQSQSQSPEGLEKQQ-----------EDEREEENL 419
             :  :.   .* :.* * :   :. :: :                        .   . :

S.cerevisiae -----------------------------
C.glabrata   TTHISISSITNSTNTLDTVKEEDMHLQKV 487
Y.lipolytica SAEASA-SAAAAYSAVDTSTDQDVEMGDA 422
C.albicans   AAETKR-------MKLDGSV--------- 432
```

**Figure S1. Multiple alignment of Opi1 polypeptides from *S. cerevisiae*, *C. glabrata, Y. lipolytica* and *C. albicans* using Clustal Omega.** The conserved domains LZ, UCR and AID are indicated in yellow, green, and light blue respectively. The PA binding region and the FFAT motif are indicated in orange and red respectively. The FFAT-like motif present in *S.cerevisiae* Opi1 is indicated in pale red. Amino acid residues referred in the text are boxed and their corresponding mutation indicated above.

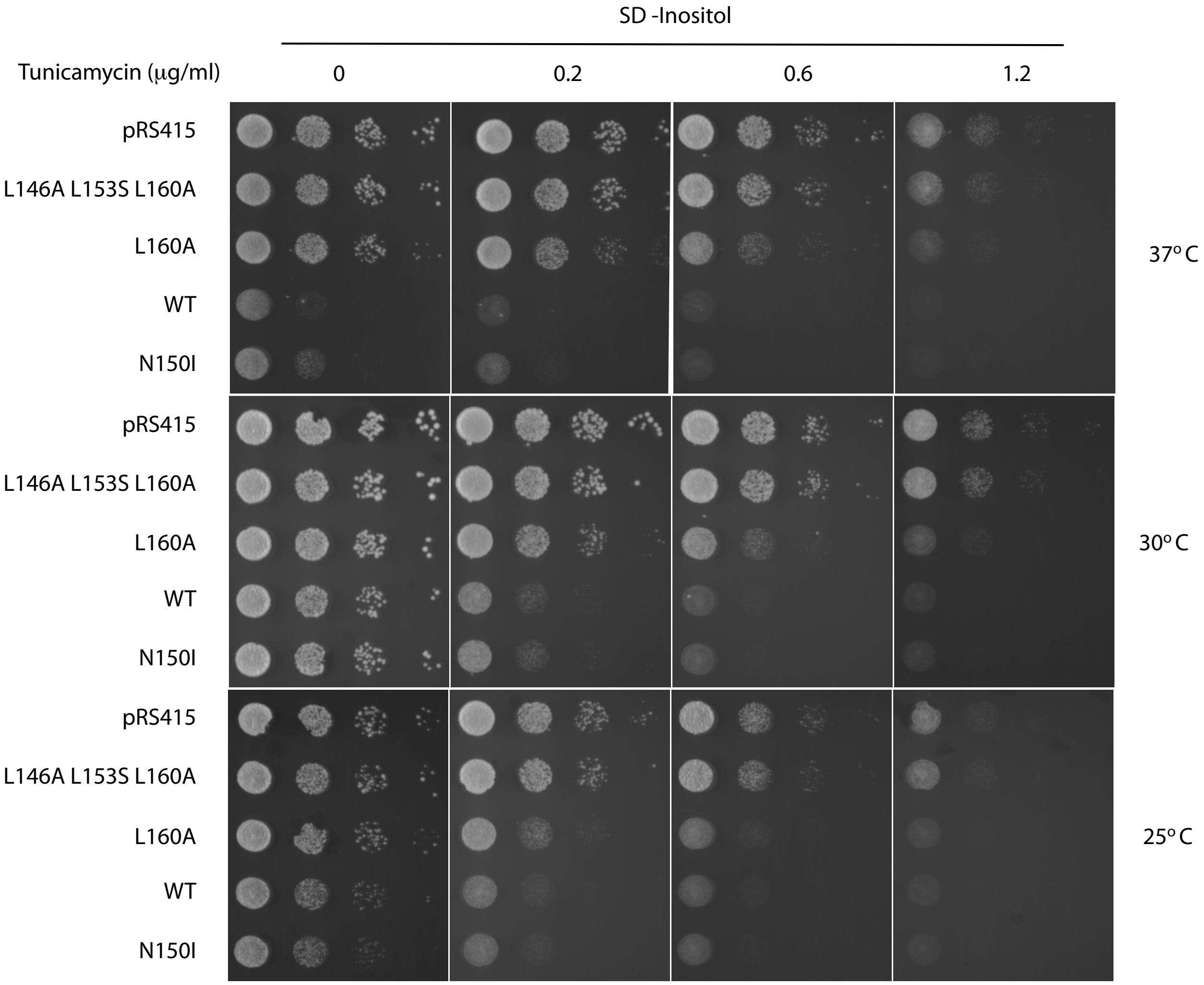


**Figure S2. Non-ionic replacement of N150 residue by isoleucine does not elicit an *ino* phenotype.** The indicated allelic variants encoded in a pRS415 vector were expressed in *opi1*Δ cells. Transformed cells were grown overnight in selective defined media containing 0.2 mM inositol. Cells were thoroughly washed and spotted as 10-fold serial dilutions at an initial cell density of $OD_{660\ nm}$ of 0.4 onto synthetic selective solid media without inositol and the indicated concentration of tunicamycin. Plates were incubated at 25°C, 30°C and 37°C for 2 days. For assessing the possible effect of N150I mutation attenuating Opi1 repression, LOF variants $Opi1^{L160A}$ and $Opi1^{L146A\ L153S\ L160A}$ were included.

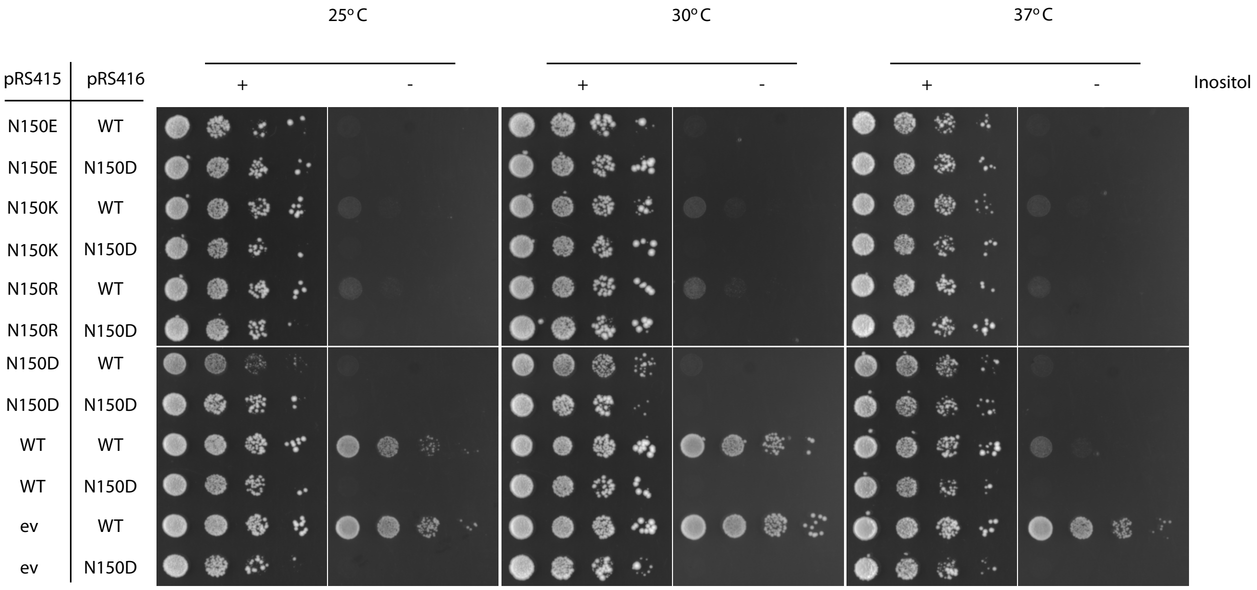


**Figure S3. Co-expression of Opi1 variants of opposite charge at residue 150 do not prevent repression.** *opi1*Δ cells transformed with a pRS416 vector expressing the WT or the N150D variant of Opi1 and a pRS415 vector encoding or not the indicated *OPI1* allelic variants, were grown in synthetic selective media containing 0.2 mM inositol for 16 hours at 30° C. Cells were thoroughly washed and spotted as a 10-fold serial dilution at an initial cell density of $OD_{660\ nm}$ of 0.4 onto synthetic selective solid media containing or not 0.2 mM inositol. Plates were incubated at the indicated temperatures for 3 days. ev, empty vector

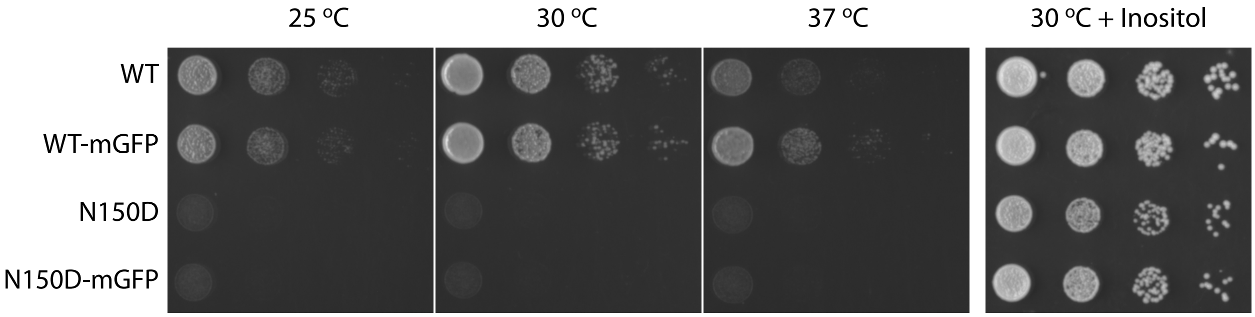


**Figure S4. C-terminal mGFP tagging does not attenuate Opi1 repression**. The indicated allelic variants encoded in a pRS415 vector were expressed in *opi1*Δ cells. Transformed cells were grown overnight in selective define media containing 0.2 mM inositol. Cells were thoroughly washed and spotted as a 10-fold serial dilution at an initial cell density of $OD_{660\ nm}$ of 0.4 onto synthetic selective solid media containing or not 0.2 mM inositol. Plates were incubated at 30° C for 2 days.

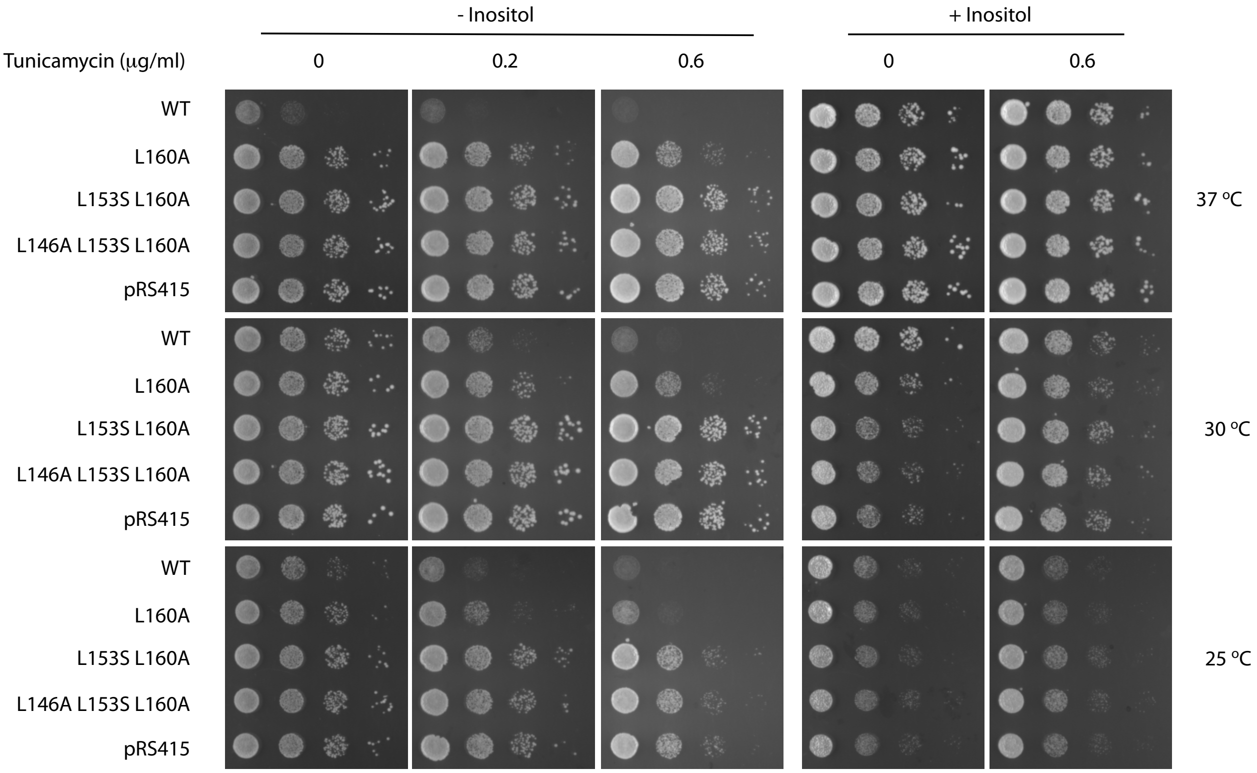


**Figure S5. Successive mutations of *d* leucine residues prevent Opi1 repression.** The indicated allelic variants encoded in a pRS415 vector were expressed in *opi1*Δ cells. Transformed cells were grown overnight in selective define media containing 0.2 mM inositol. Cells were thoroughly washed and spotted as a 10-fold serial dilution at an initial cell density of $OD_{660\ nm}$ of 0.4 onto synthetic selective solid media containing or not 0.2 mM inositol and the indicated concentration of tunicamycin. Plates were incubated at 25° C, 30° C and 37 ° C for 2 days.

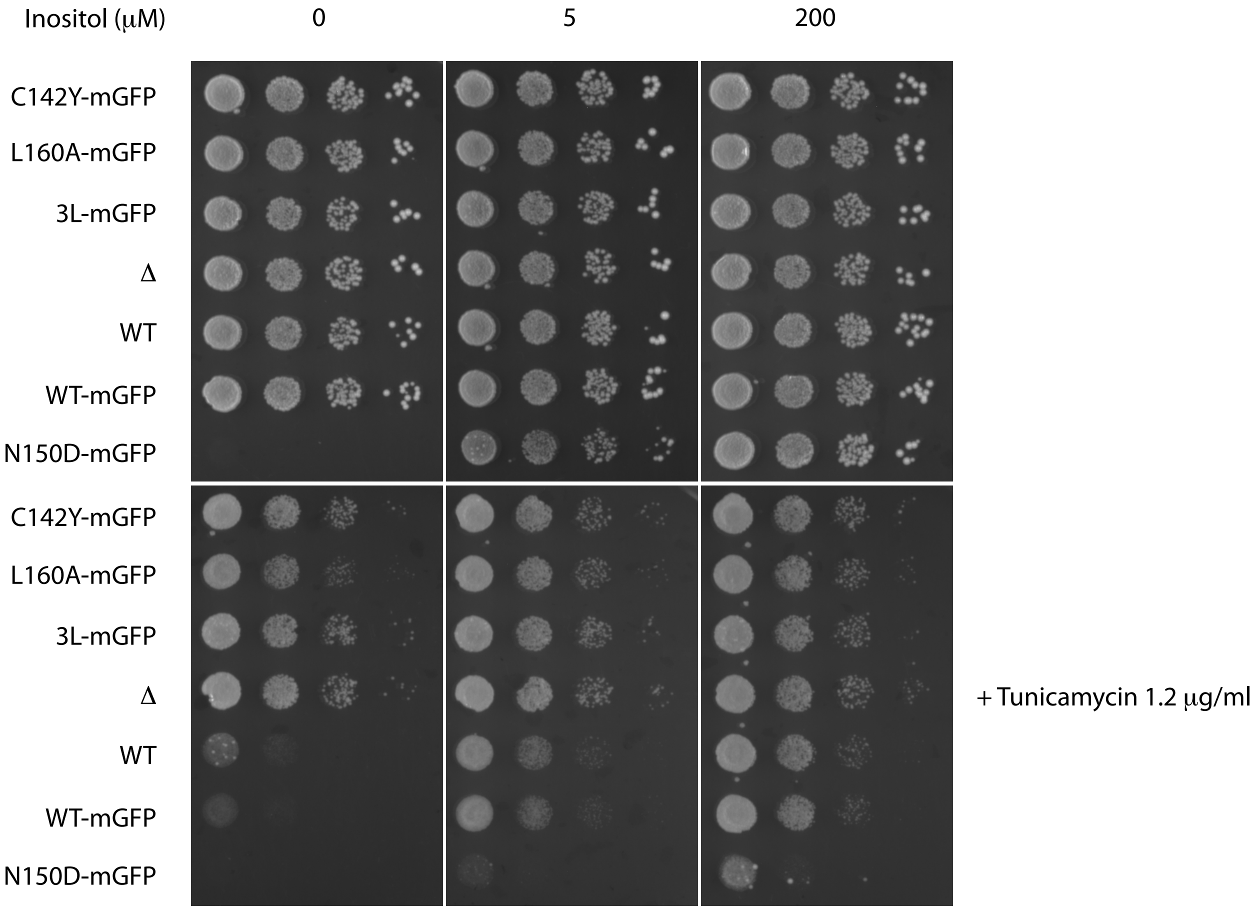


**Figure S6. Phenotypic spectrum of LZ mutations.** Yeast haploid cells expressing the indicated *OPI1* allelic variants from *OPI1* chromosomal locus were grown overnight in defined media containing 0.2 mM inositol. Cells were thoroughly washed and spotted as a 10-fold serial dilution at an initial cell density of $OD_{660\ nm}$ of 0.4 onto synthetic solid media containing or not the indicated amount of inositol +/- tunicamycin. Plates were incubated at 30°C for 2 days.

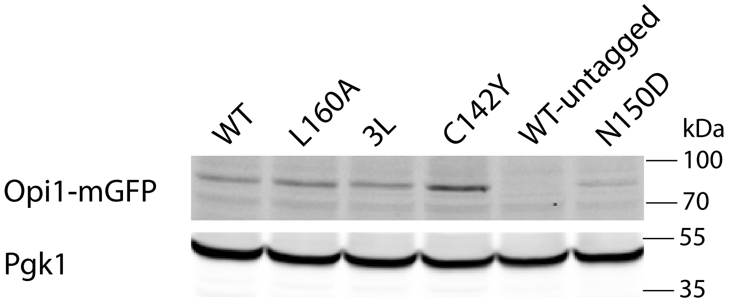


**Figure S7. Steady state levels of LZ Opi1-mGFP variants**. Haploids cells grown on synthetic define media containing 0.2 mM inositol were cultivated for 8 hours on synthetic define media containing 5 µM inositol. Whole cell extracts were prepared and analyzed by SDS-PAGE. Abundance of Opi1-mGFP polypeptide was estimated by western blotting against GFP. Loading standardization was based on immunodetection of Pgk1.

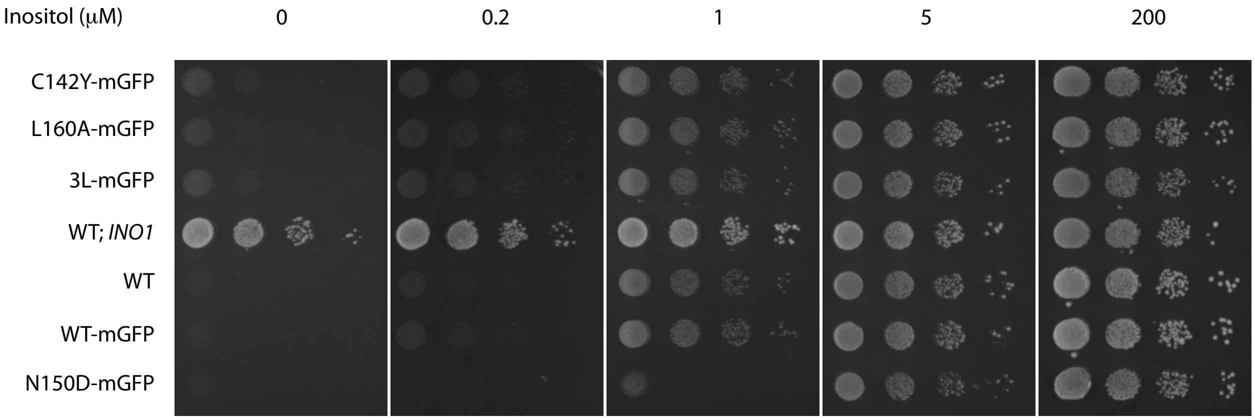


**Figure S8. Inositol dependent growth phenotype of *ino1*Δ strains.** Yeast *ino1*Δ cells expressing the indicated *OPI1* allelic variants from *OPI1* chromosomal locus were grown overnight in defined media containing 0.2 mM inositol. Cells were thoroughly washed and spotted as a 10-fold serial dilution at an initial cell density of $OD_{660\ nm}$ of 0.4 onto synthetic selective solid media containing the indicated amount of inositol. Plates were incubated at 30°C for 2 days.

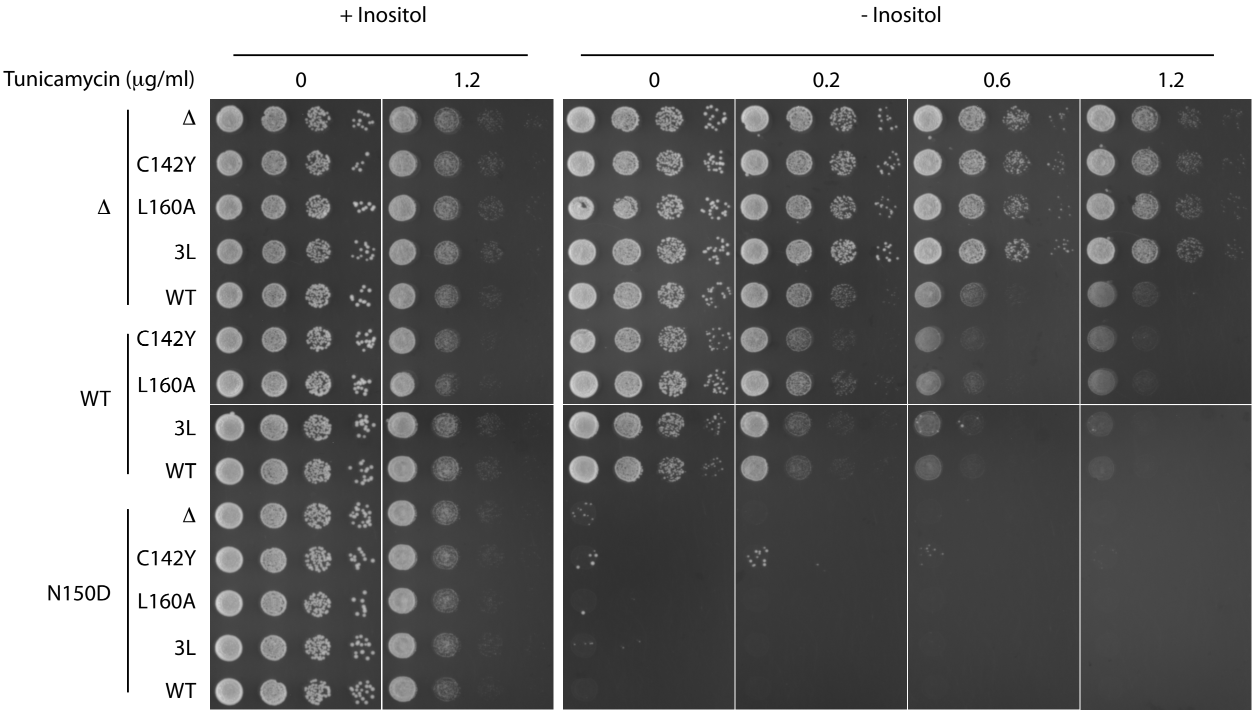


**Figure S9. Genetic analysis for Opi1$^{N150D}$ variant.** Diploid cells expressing the indicated LZ allelic variants C-terminally tagged with -mGFP from *OPI1* chromosomal loci were grown overnight in synthetic media containing 0.2 mM inositol at 30° C. Cells were thoroughly washed and spotted as a 10-fold serial dilution at an initial cell density of $OD_{660\ nm}$ of 0.4 onto synthetic solid media containing or not 0.2 mM inositol and the indicated amounts of tunicamycin. Plates were incubated at 30° C for 2 days.

A

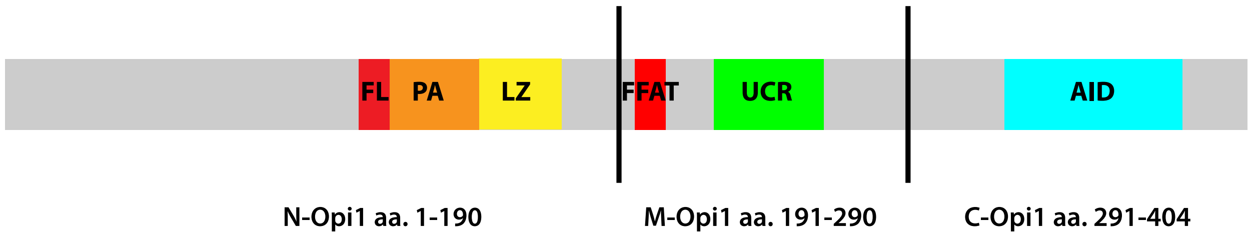


B

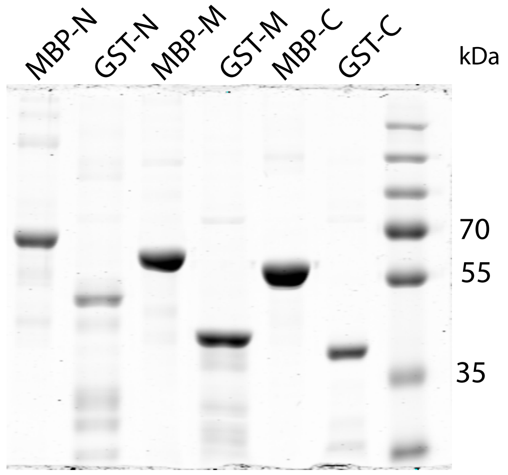


C

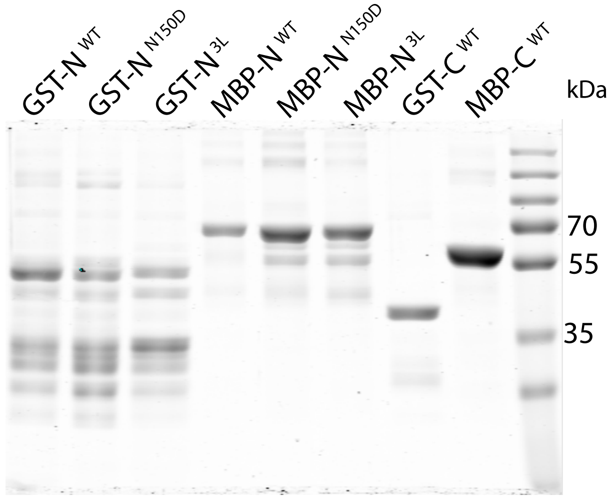


D

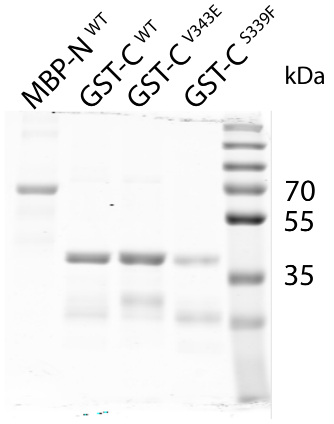


**Figure S10. Affinity purified preparations of N-terminally tagged recombinant Opi1 peptides.** **(A)** Opi1 polypeptide was dissected in the three indicated regions and cloned C-terminally of

MBP and GST. The indicated Opi1 chimeras were expressed in bacteria and affinity purified. Protein preparations were analyzed by SDS-PAGE and stained with Coomassie Brilliant Blue. Relevant molecular weight markers are indicated. (**B)** MBP and GST Opi1 N-, M- and C- terminal peptides. **(C, D)** MBP and GST Opi1 N- and C- terminal peptides for wild type compared to various mutants preparations.

## Table S1: Strains

| Strain | Relevant genotype | Source |
|---|---|---|
| BY 4742 | MAT $\alpha$ *his3*Δ1 *leu2*Δ0 *lys2*Δ0 *ura3*Δ0 | Euroscarf |
| PMY704 | BY4742 *opi1*Δ::HYG *scs2*Δ::NAT *scs22*Δ::KAN | This study |
| PMY593 | BY4741 *OPI1*-GFP::*HIS3* | Invitrogen |
| PMY1111 | BY4743 *OPI1*/ *opi1*Δ::KAN | Euroscarf |
| PMY1113 | BY4742 *opi1*Δ::KAN | This study |
| PMY294 | BY4742 *nte1*Δ::NAT | Lab collection |
| PMY454 | BY4741 *ino1*Δ::KAN | Euroscarf |
| PMY1141 | BY4742 *OPI1*-mGFP | This study |
| PMY1145 | BY4742 *OPI1*-mGFP (L146A L153S L160A) | This study |
| PMY1129 | BY4742 *OPI1*-mGFP (C142Y) | This study |
| PMY1133 | BY4742 *OPI1*-mGFP (L160A) | This study |
| PMY1138 | BY4742 *OPI1*-mGFP (N150D) | This study |
| PMY1224 | *ino1*Δ::KAN *OPI1*-mGFP (C142Y) | This study |
| PMY1226 | *ino1*Δ::KAN *OPI1*-mGFP (L160A) | This study |
| PMY1229 | *ino1*Δ::KAN *OPI1*-mGFP | This study |
| PMY1231 | *ino1*Δ::KAN *OPI1*-mGFP (L146A L153S L160A) | This study |
| PMY1243 | *ino1*Δ::KAN *OPI1*-mGFP (N150D) | This study |
| PMY1168 | BY4743 *opi1*Δ::KAN / *opi1*Δ::KAN | This study |
| PMY1169 | BY4743 *opi1*Δ::KAN / *OPI1*-mGFP | This study |
| PMY1170 | BY4743 *opi1*Δ::KAN / *OPI1*-mGFP (L146A L153S L160A) | This study |
| PMY1171 | BY4743 *opi1*Δ::KAN / *OPI1*-mGFP (C142Y) | This study |
| PMY1201 | BY4743 *opi1*Δ::KAN / *OPI1*-mGFP (L160A) | This study |
| PMY1203 | BY4743 *OPI1*-mGFP / *OPI1*-mGFP (L160A) | This study |
| PMY1155 | BY4743 *OPI1*-mGFP / *OPI1*-mGFP | This study |
| PMY1167 | BY4743 *OPI1*-mGFP / *OPI1*-mGFP (C142Y) | This study |
| PMY1163 | BY4743 *OPI1*-mGFP / *OPI1*-mGFP (L146A L153S L160A) | This study |
| PMY1159 | BY4743 *OPI1*-mGFP / *OPI1*-mGFP (N150D) | This study |
| PMY1177 | BY4743 *OPI1*-mGFP (N150D) / *OPI1*-mGFP (L146A L153S L160A) | This study |
| PMY1178 | BY4743 *OPI1*-mGFP (N150D) / *OPI1*-mGFP (C142Y) | This study |
| PMY1202 | BY4743 *OPI1*-mGFP (N150D) / *OPI1*-mGFP (L160A) | This study |
| PMY1104 | BY4742 *OPI1*-mGFP (Y127D)::NAT | (Hofbauer, Gecht et al. 2018) |
| MK1017 | BRS1001 *OPI1* (S339F) | (Kaadige and Lopes 2006) |
| MK1029 | BRS1001 *OPI1* (V343E) | (Kaadige and Lopes 2006) |
| DBY12218 | S288C *HAP1 met4*Δ::KAN *OPI1* (K226E) | (Hickman, Petti et al. 2011) |
| DBY12219 | S288C *HAP1 met4*Δ::NAT *OPI1* (L255S) | (Hickman, Petti et al. 2011) |

## Table S2: Primers

| Primers | Sequence | Description |
|---|---|---|
| 5’-U-*OPI1-Sal*I | TGTCGACACGCTTACGCAGACATCTCAT | Cloning 5’ region of *OPI1* orf |
| 5’-D-*OPI1-Xma*I | TCCCGGGCATCAATGACTAGTATCTTCG | Cloning 5’ region of *OPI1* orf |
| 3’-U-*OPI1-Xma*I | TCCCGGGGGACTAACCGAGACAGATT | Cloning 3’ region of *OPI1* orf |
| 3’-D-*OPI1-Sac*I | TGAGCTCCTTGTGTTGGTAATTGGTTTC | Cloning 3’ region of *OPI1* orf |
| *Asc*I-3’-Up-*OPI1* | ATGGCGCGCCGAGACAGATTGAGGTCTTTC | Cloning 3’ region of *OPI1* orf |
| 5’-*OPI1*-M | AAAGCATATCAGGCCAGAACG | Cloning of *OPI1* orf |
| 3’-*OPI1*-M | AAGCGGGGCTGGTACAATAT | Cloning of *OPI1* orf |
| F2-G | ATGGGAAAGTACTCATTCTC | Cloning 3’-*OPI1*-GFP |
| 3’-GFP-HIS3-*Sac*I | AGAGCTCCTGTCAAGGAGGGTATTCTG | Cloning 3’-*OPI1*-GFP |
| mGFP-A206Kf | CATTACCTGTCCACACAATCTAAACTTTCGAAAGATCCCAACG | Mutagenesis |
| mGFP-A206Kr | CGTTGGGATCTTTCGAAAGTTTAGATTGTGTGGACAGGTAATG | Mutagenesis |
| 5’-H144L | TTGTAACGTGCTTGCTTCTTTTAAAGCTGG | Mutagenesis |
| 3’-H144L | CCAGCTTTAAAAGAAGCAAGCACGTTACAA | Mutagenesis |
| 5’-N150D | CTTTTAAAGCTGGCCGATAAGCAGCTTTCCGATAA | Mutagenesis |
| 3’-N150D | TTATCGGAAAGCTGCTTATCGGCCAGCTTTAAAAG | Mutagenesis |
| 5’-N150I | CTTTTAAAGCTGGCCATTAAGCAGCTTTCCGATAA | Mutagenesis |
| 3’-N150I | TTATCGGAAAGCTGCTTAATGGCCAGCTTTAAAAG | Mutagenesis |
| 5’-N150E | CTTTTAAAGCTGGCCGAAAAGCAGCTTTCCGATAA | Mutagenesis |
| 3’-N150E | TTATCGGAAAGCTGCTTTTCGGCCAGCTTTAAAAG | Mutagenesis |
| 5’-N150K | CTTTTAAAGCTGGCCAAAAAGCAGCTTTCCGATAA | Mutagenesis |
| 3’-N150K | TTATCGGAAAGCTGCTTTTTGGCCAGCTTTAAAAG | Mutagenesis |
| 5’-N150R | CTTTTAAAGCTGGCCAGAAAGCAGCTTTCCGATAA | Mutagenesis |
| 3’-N150R | TTATCGGAAAGCTGCTTTCTGGCCAGCTTTAAAAG | Mutagenesis |
| 5’-L146A | GTAACGTGCTTGCATCTTGCAAAGCTGGCCAATAAGC | Mutagenesis |
| 5’-L146A | GCTTATTGGCCAGCTTTGCAAGATGCAAGCACGTTAC | Mutagenesis |
| 5’-L153S | GCTGGCCAATAAGCAGTCTTCCGATAAAATCTCG | Mutagenesis |
| 3’-L153S | CGAGATTTTATCGGAAGACTGCTTATTGGCCAGC | Mutagenesis |
| 5’-N-*EcoR*I | ATGAATTCATGTCTGAAAATCAACGTTTAG | Cloning N-Opi1 (pMAL and pGEX vectors) |
| 3’-N-*Not*I | ATGCGGCCGCTGTCTCGTCCTCGCCAGC | Cloning N-Opi1 (pGEX vector) |
| 3’-N-*Sal*I | ATGTCGACTTATGTCTCGTCCTCGCCAGC | Cloning N-Opi1 (pMAL vector) |
| 5’-M-*EcoR*I | ATGAATTCTCGTCAGACGAAGACGAC | Cloning M-Opi1 (pMAL and pGEX vectors) |
| 3’-M-*Not*I | ATGCGGCCGCTTGCTGTTGCTGTTGCTC | Cloning M-Opi1 (pGEX vector) |
| 3’-M-*Sal*I | ATGTCGACTTATTGCTGTTGCTGTTGCTC | Cloning M-Opi1 (pMAL vector) |
| 5’-C-*EcoR*I | ATGAATTCCAGCAACAGCAGCAGCAG | Cloning C-Opi1 (pMAL and pGEX vectors) |
| 3’-C-*Not*I | ATGCGGCCGCGTCCTTGCTATCCAGGTTG | Cloning C-Opi1 (pGEX vector) |
| 3’-C-*Xba*I | ATTCTAGATTAGTCCTTGCTATCCAGGTTG | Cloning C-Opi1 (pMAL vector) |
| 5’-*OPI1*g | CTCACAGTACAACAGCGACG | Genotyping |
| 3’-*OPI1*g | TACAGTTCTCCCGTGGTCAC | Genotyping |

## Table S3: Plasmids

| Plasmid | Description | Source |
|---|---|---|
| pPM169 | pRS415-*OPI1* | This study |
| pPM170 | pRS415-5'/3' flanking *OPI1* ORF sequences | This study |
| pRS416-*NTE1*<br>pRS415-*NTE1* | *NTE1* gene under its own promoter on a pRS416 or pRS415 vector | (Fernandez-Murray, Gaspard et al. 2009) |
| pPSB2F | pRS415-*OPI1* (L143S) | This study |
| pPM24 | pRS415-*OPI1* (H144L) | This study |
| pPSB27 | pRS415-*OPI1* (H144Y) | This study |
| pPSB2J | pRS415-*OPI1* (K147E) | This study |
| pPM33 | pRS415-*OPI1* (N150D) | This study |
| pPM245 | pRS415-*OPI1* (N150E) | This study |
| pPM246 | pRS415-*OPI1* (N150I) | This study |
| pPM247 | pRS415-*OPI1* (N150K) | This study |
| pPM248 | pRS415-*OPI1* (N150R) | This study |
| pPM449 | pRS415-*OPI1* (K226E) | This study |
| pPM450 | pRS415-*OPI1* (N150D K226E) | This study |
| pPM452 | pRS415-*OPI1* (L252F) | This study |
| pPM451 | pRS415-*OPI1* (N150D L252F) | This study |
| pPM455 | pRS415-*OPI1* (L255S) | This study |
| pPM454 | pRS415-*OPI1* (N150D L255S) | This study |
| pPM390 | pRS415-*OPI1* (S339F) | This study |
| pPM399 | pRS415-*OPI1* (N150D S339F) | This study |
| pPM391 | pRS415-*OPI1* (V343E) | This study |
| pPM384 | pRS415-*OPI1* (N150D V343E) | This study |
| pPM62 | pRS416-*OPI1* (N150D) | This study |
| pPM108 | pRS415-*OPI1* (L153S L160A) | This study |
| pPM112 | pRS415-*OPI1* (L146A L153S L160A) | This study |
| pPM494 | pRS415-*OPI1*-mGFP; orf flanked by upstream and downstream regions of *OPI1* locus, for healing a double strand DNA break introduced at *opi1*Δ::kanMX4 | This study |
| pPM500 | pRS415-*OPI1*-mGFP (L160A), analogous to pPM494 | This study |
| pPM501 | pRS415-*OPI1*-mGFP (N150D), analogous to pPM494 | This study |
| pPM502 | pRS415-*OPI1*-mGFP (L146A L153S L160A), analogous to pPM494 | This study |
| pPM503 | pRS415-*OPI1*-mGFP (C142Y), analogous to pPM494 | This study |
| pPM293 | pGEX-N-*OPI1* | This study |
| pPM297 | pMAL-N-*OPI1* | This study |
| pPM305 | pGEX-M-*OPI1* | This study |
| pPM313 | pMAL-M-*OPI1* | This study |
| pPM307 | pGEX-C-*OPI1* | This study |
| pPM307 | pMAL-C-*OPI1* | This study |
| pPM321 | pGEX-N-*OPI1* (N150D) | This study |
| pPM323 | pMAL-N-*OPI1* (N150D) | This study |
| pPM322 | pGEX-N-*OPI1* (L146A L153S L160A) | This study |
| pPM324 | pMAL-N-*OPI1* L146A L153S L160A) | This study |
| pPM341 | pGEX-C-*OPI1* (V343E) | This study |
| pPM307 | pGEX-C-*OPI1*(S339F) | This study |
| pCW4 | ARS CEN *LEU2 OPI1* (L160A) | (Wagner, Blank et al. 1999) |
| pPM41 | pRS200-*OPI1* (C142Y) | (Kaadige and Lopes 2006) |
| pPM42 | pRS200-*OPI1* (L252F) | (Kaadige and Lopes 2006) |
| pPM551 | p2UG-2XORE-Luc; firefly luciferase gene under the promoter of *FOX3* gene | (Phelps, Gburcik et al. 2006) |
| pAR1275 | pRS425 carrying Cas9 and a guide RNA targeting KAN gene marker | Dr. Adam Rudner |

Table S4: LC mobile phase composition and gradient

| Time (min) | Flow rate (ml/min) | Line A Acetonitrile<br><br>% | Line B Isopropanol<br><br>% | Line C Water<br><br>% | Line D 0.1% formic acid + 0.4 M ammonium formate<br>% |
|---|---|---|---|---|---|
| 0 | 0.2 | 43.6 | 26.3 | 27.6 | 2.5 |
| 3 | 0.2 | 43.6 | 26.3 | 27.6 | 2.5 |
| 8 | 0.2 | 37.1 | 37.7 | 22.6 | 2.5 |
| 9 | 0.2 | 33.6 | 43.9 | 20.0 | 2.5 |
| 18 | 0.2 | 13.7 | 79.0 | 4.8 | 2.5 |
| 26 | 0.2 | 9.3 | 86.9 | 1.4 | 2.5 |
| 30 | 0.2 | 9.3 | 86.9 | 1.4 | 2.5 |
| 35 | 0.2 | 43.6 | 26.3 | 27.6 | 2.5 |
| 45 | 0 | | | | |

Table S5: Q Exactive acquisition parameter

| | Positive ion mode | Negative ion mode |
|---|---|---|
| **Tune settings** | | |
| Spray voltage | 3400 (+) | 3200 (-) |
| Capillary temperature | 300 | 300 |
| Sheath gas | 40 | 40 |
| Aux gas | 2 | 2 |
| Spare gas | 0 | 0 |
| Probe heater temp | 350 | 350 |
| S-lens RF lewvel | 50 | 50 |
| Ion source | HESI | HESI |
| **Global setting** | | |
| Use lock masses | Best | Best |
| Chrom. Peak width (FWHM) | 15 s | 15 s |
| Method duration | 45 min | 45 min |
| **Experiment** | | |
| *Full MS/dd-MS$^2$ (TOPN)* | | |
| Runtime | 0 to 45 min | 0 to 45 min |
| Polarity | Positive | Negative |
| Microscans | 1 | 1 |
| Resolution | 70,000 | 70,000 |
| AGC target | 1e6 | 1e6 |
| Maximun IT | 246 ms | 246 ms |
| Number of scan ranges | 1 | 1 |
| Scan range | 300 to 1700 m/z | 300 to 1700 m/z |
| Spectrum data type | Profile | Profile |
| *dd-MS$^2$* | | |
| Microscans | 1 | 1 |
| Resolution | 17,500 | 17,500 |
| AGC target | 5e5 | 5e5 |
| Maximum IT | 150 ms | 150 ms |
| Loop count | 15 | 15 |
| Top N | 15 | 15 |
| Isolation window | 1 m/z | 1 m/z |
| Sacn range | 200 to 2000 m/z | 200 to 2000 m/z |
| (N)CE / stepped (N)CE | nce: 25, 35 | nce: 25, 35 |

| | | |
|---|---|---|
| Spectrum data type | Profile | Profile |
| *dd settings* | | |
| Minimum AGC target | 1e4 | 5e3 |
| Intensity threshold | 6.7e4 | 3.3e4 |
| Peptide match | Off | Off |
| Exclude isotopes | On | On |
| Dynamic exclusion | 8 s | 8 s |
| If idle | Pick others | Pick others |

## Table S6: Lipid identification and quantitation parameters

| | |
|---|---|
| Database name | General-lcms-product |
| Search type | Product (LC-MS) |
| Precursor tol | 5 |
| Precursor tol type | ppm |
| Product tol | 8 |
| Product tol type | ppm |
| Product threshold | 1 |
| Product threshold type | relative |
| Group | disable |
| Merge type | Mean |
| Filter type | Setting filter |
| Merge Rt tolerance | 0.05 |
| Merge Rt Crr tolerance | 0.5 |
| Merge Sn threshold | 3 |
| Merge Ity ratio threshold | 1.5 |
| Merge valid Cnt ratio threshold | 0.5 |
